# BTK drives neutrophil activation for sterilizing antifungal immunity

**DOI:** 10.1172/JCI176142

**Published:** 2024-05-02

**Authors:** Jigar V. Desai, Marissa A. Zarakas, Andrew L. Wishart, Mark Roschewski, Mariano A. Aufiero, Agnes Donkò, Gustaf Wigerblad, Neta Shlezinger, Markus Plate, Matthew R. James, Jean K. Lim, Gulbu Uzel, Jenna R.E. Bergerson, Ivan Fuss, Robert A. Cramer, Luis M. Franco, Emily S. Clark, Wasif N. Khan, Daisuke Yamanaka, Georgios Chamilos, Jamel El-Benna, Mariana J. Kaplan, Louis M. Staudt, Thomas L. Leto, Steven M. Holland, Wyndham H. Wilson, Tobias M. Hohl, Michail S. Lionakis

**Affiliations:** 1 Fungal Pathogenesis Section, Laboratory of Clinical Immunology & Microbiology (LCIM), National Institute of Allergy & Infectious Diseases (NIAID), National Institutes of Health (NIH), Bethesda, Maryland, USA.; 2 Lymphoid Malignancies Branch, National Cancer Institute, NIH, Bethesda, Maryland, USA.; 3 Louis V. Gerstner, Jr. Graduate School of Biomedical Sciences, Memorial Sloan Kettering Cancer Center, New York, New York, USA.; 4 Molecular Defenses Section, LCIM, NIAID, NIH, Bethesda, Maryland, USA.; 5 Systemic Autoimmunity Branch, National Institute of Arthritis and Musculoskeletal and Skin Diseases (NIAMS), NIH, Bethesda, Maryland, USA.; 6 Infectious Disease Service, Department of Medicine, Memorial Sloan Kettering Cancer Center, New York, New York, USA.; 7 Department of Microbiology and Immunology, Geisel School of Medicine at Dartmouth, Hanover, New Hampshire, USA.; 8 Department of Microbiology, Icahn School of Medicine at Mount Sinai, New York, New York, USA.; 9 Immunopathogenesis Section, LCIM, NIAID, NIH, Bethesda, Maryland, USA.; 10 Primary Immune Deficiency Clinic, LCIM, NIAID, NIH, Bethesda, Maryland, USA.; 11 Mucosal Immunity Section, LCIM, NIAID, NIH, Bethesda, Maryland, USA.; 12 Functional Immunogenomics Section, NIAMS, NIH, Bethesda, Maryland, USA.; 13 Department of Microbiology and Immunology, Miller School of Medicine, University of Miami, Miami, Florida, USA.; 14 Laboratory for Immunopharmacology of Microbial Products, School of Pharmacy, Tokyo University of Pharmacy and Life Sciences, Tokyo, Japan.; 15 Department of Clinical Microbiology and Microbial Pathogenesis, University Hospital of Heraklion, Heraklion, Crete, Greece.; 16 Centre de Recherche sur l’Inflammation, Laboratoire d’Excellence Inflamex, Faculté de Médecine Xavier Bichat, Université de Paris-Cité, INSERM-U1149, CNRS-ERL8252, Paris, France.; 17 Human Oncology and Pathogenesis Program, Memorial Sloan Kettering Cancer Center, New York, New York, USA.

**Keywords:** Immunology, Infectious disease, Fungal infections, Innate immunity, Neutrophils

## Abstract

We describe a previously unappreciated role for Bruton’s tyrosine kinase (BTK) in fungal immune surveillance against aspergillosis, an unforeseen complication of BTK inhibitors (BTKi) used for treating B cell lymphoid malignancies. We studied BTK-dependent fungal responses in neutrophils from diverse populations, including healthy donors, patients who were treated with BTKi, and X-linked agammaglobulinemia patients. Upon fungal exposure, BTK was activated in human neutrophils in a TLR2-, Dectin-1-, and FcγR-dependent manner, triggering the oxidative burst. BTK inhibition selectively impeded neutrophil-mediated damage to *Aspergillus* hyphae, primary granule release, and the fungus-induced oxidative burst by abrogating NADPH oxidase subunit p40^phox^ and GTPase RAC2 activation. Moreover, neutrophil-specific *Btk* deletion in mice enhanced aspergillosis susceptibility by impairing neutrophil function, not recruitment or lifespan. Conversely, GM-CSF partially mitigated these deficits by enhancing p47^phox^ activation. Our findings underline the crucial role of BTK signaling in neutrophils for antifungal immunity and provide a rationale for GM-CSF use to offset these deficits in patients who are susceptible.

## Abstract 2

BTK inhibition impairs neutrophil antifungal effector functions, resulting in susceptibility to invasive aspergillosis.

## Graphical abstract

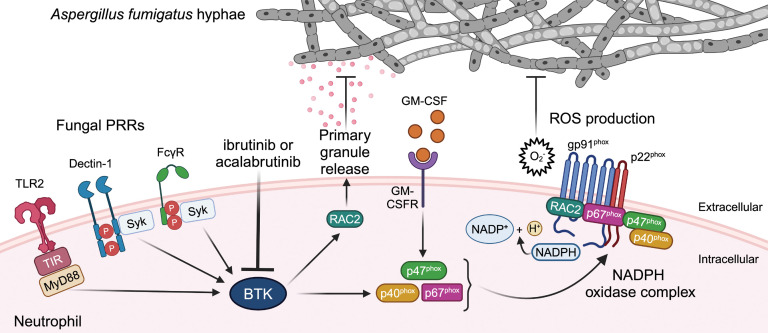

## Introduction

Invasive aspergillosis (IA), most often caused by the ubiquitous inhaled mold *Aspergillus fumigatus*, is an opportunistic fungal infection that exploits numeric or functional neutrophil defects (1–3). IA affects over 200,000 patients per year worldwide and leads to a high mortality rate despite contemporary antifungal therapies (1–3). Recently, IA has emerged as an unanticipated complication of Bruton’s tyrosine kinase inhibitor (BTKi) treatment, implying a critical role for BTK in fungal immune surveillance (4–10).

BTK is well known for its critical roles in B cell differentiation, proliferation, and function by integrating signals from several cell surface receptors including the B cell receptor (BCR) (11, 12). Inherited *BTK* deficiency causes the primary immunodeficiency disorder, X-linked agammaglobulinemia (XLA, OMIM no. 300755), first described by Bruton in 1952 (13). XLA patients have markedly decreased numbers of mature B cells and markedly reduced levels of serum antibodies and immunoglobulins (14, 15). As a result, they are susceptible to infections by a variety of bacteria and enteroviruses for which antibodies are critical in host defense (14, 15).

In recent years, the pharmacological targeting of constitutively active BCR signaling in malignant B cells with BTKi (i.e., ibrutinib, acalabrutinib, and zanubrutinib), has shifted the paradigm for the treatment of several B cell lymphoid malignancies (4, 16–20). As with patients with XLA, bacterial and viral infections have been reported in patients who were treated with BTKi (5, 17). Surprisingly, patients receiving BTKi have also exhibited a heightened risk for life-threatening invasive fungal infections, primarily aspergillosis (4–10, 21), with such infections being infrequently reported in patients with XLA (22, 23). These observations suggest a critical, yet unforeseen role for BTK in orchestrating human antifungal immune responses, the mechanistic basis of which remains poorly defined (24–29).

Here, we studied BTK-dependent neutrophil responses against *A*. *fumigatus* in diverse human populations with inherited BTK deficiency and pharmacological BTK blockade and in a mouse model of aspergillosis. We identify the fungal-sensing receptors that act as upstream activators of BTK and characterize the anti-*Aspergillus* neutrophil functions that selectively rely on BTK signaling and the BTK-regulated intracellular effector molecules that modulate these functions. We show that GM-CSF partially rescues the BTKi-induced neutrophil functional defects in vitro and in vivo, thereby providing a translational strategy to overcome BTKi-driven impairment in innate protection during fungal infection. Our findings provide insights into the mechanistic role of BTK in neutrophil-mediated antifungal defense and help explain why patients treated with BTKi develop IA.

## Results

### Neutrophil-specific Btk ablation confers murine susceptibility to pulmonary aspergillosis.

**Figure 1:**
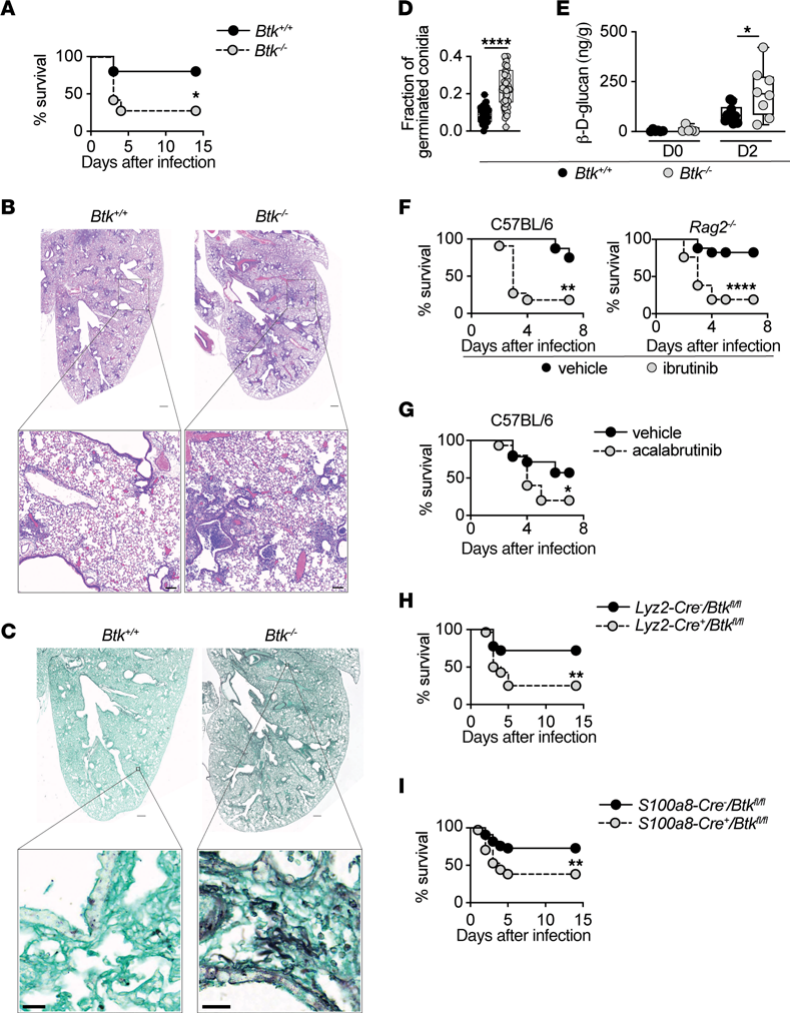
Neutrophil-specific BTK confers protection during pulmonary aspergillosis. (**A**) Survival of WT and *Btk^–/–^* mice after infection with *A*. *fumigatus* (*n* = 10–14). (**B** and **C**) Representative micrographs of (**B**) H&E-stained and (**C**) Grocott’s methenamine silver–stained (GMS-stained) lung sections at day 4 after infection. Scale bars: 1 mm (upper panels, **B** and **C**), 250 μm (lower panel, **B**), 25 μm (lower panel, **C**) (*n* = 4). (**D**) Quantification of the proportion of germinating *A*. *fumigatus* conidia in GMS-stained lung sections. Each dot depicts an individual affected region of the lung; 4 such areas were randomly chosen per mouse (*n* = 4 mice) and germinated conidia were enumerated. (**E**) β-D-glucan levels in lung homogenates at steady state and day 2 after infection. Each dot depicts an individual mouse (*n* = 5–10). (**F**) Survival of ibrutinib- or vehicle-treated WT and *Rag2^–/–^* mice after infection with *A*. *fumigatus* (*n* = 11–21). (**G**–**I**) Survival of the indicated mice after infection with *A*. *fumigatus* (**G**, *n* = 14–15, **H**, *n* = 18–28; **I**, *n* = 33–34). Box and whisker plots depict values ranging from minimum to maximum (**D** and **E**). *P<0.05, **P<0.01, ****P<0.0001, determined using log-rank test (**A** and **F**–**I**), 2-sided Mann-Whitney *U* test (**D** and **E**), or 2-sided unpaired *t* test (**E**).

To examine the role of BTK in anti-*Aspergillus* host defense in vivo, we infected WT and *Btk^–/–^* mice with *A*. *fumigatus* conidia via pharyngeal aspiration. Consistent with our observation (4), *Btk^–/–^* mice were susceptible to pulmonary aspergillosis, as 71% of them succumbed within 4 days of fungal inoculation, whereas mortality in WT mice was 20% (Figure 1A). Histological examination revealed greater inflammatory foci, tissue injury, and fungal invasion throughout the lung parenchyma of *Aspergillus*-infected *Btk^–/–^* mice compared with WT controls (Figure 1, B and C). We also observed a significantly greater proportion of fungal conidia germinating into the tissue-invasive hyphal morphotype in the lungs of *Btk^–/–^* mice (Figure 1D). Consistent with this observation, we detected higher levels of the fungal cell wall polysaccharide β-glucan in the lungs of *Btk^–/–^* mice compared with WT controls (Figure 1E).

Treatment of WT mice with the BTKi ibrutinib or acalabrutinib — a second-generation BTKi that features greater selectivity for BTK and lacks ITK or TEC targeting (18, 30) — phenocopied the increased susceptibility to pulmonary aspergillosis observed in *Btk^–/–^* mice (Figure 1, A, F, and G). Ibrutinib-treated mice exhibited a greater proportion of conidia germinating into hyphae in the lung than control mice (Supplemental Figure 1; supplemental material available online with this article; https://doi.org/10.1172/JCI176142DS1). BTK is highly expressed in B cells and regulates their development, differentiation, proliferation, and function (11, 12). However, B cells are not essential for protection against IA in humans or mice (31, 32). Consistent with these observations, ibrutinib treatment of *Aspergillus*-infected lymphocyte-deficient *Rag2^–/–^* mice led to an increase in mortality similar to that observed in ibrutinib-treated, lymphocyte-sufficient WT mice (Figure 1F). These data indicate that BTK inhibition impairs anti-*Aspergillus* host defense by exerting detrimental effects on nonlymphoid cells.

Myeloid phagocytes, including neutrophils and CCR2^+^ inflammatory monocytes, are critical orchestrators of the pulmonary anti-*Aspergillus* defense (31, 33–35). Beyond B cells, BTK is expressed in myeloid phagocytes, including neutrophils (36), which are the principal immune effector cells that promote fungal clearance (2, 31, 32, 37–40). Thus, to directly assess whether BTK-dependent protection against aspergillosis relies on BTK expression in myeloid phagocytes, we generated mice with myeloid phagocyte-specific conditional ablation of *Btk* by crossing *Btk^fl/fl^* with *Lyz2-Cre^tg^* mice. *Lyz2-Cre/Btk^fl/fl^* mice were susceptible to aspergillosis (75% mortality versus 28% in littermate control mice; Figure 1H), underscoring the critical contribution of BTK-expressing myeloid phagocytes in anti-*Aspergillus* protection. We then asked whether neutrophil-specific *Btk* ablation also heightens murine susceptibility to aspergillosis. Therefore, we crossed *Btk^fl/fl^* with *S100a8-Cre^tg^* mice and found that *S100a8-Cre/Btk^fl/fl^* mice exhibited significantly greater mortality after infection (62% mortality versus 27% in littermate control mice; Figure 1I). Taken together, these data show that BTK promotes protection against pulmonary aspergillosis by exerting direct effects on myeloid phagocytes and primarily neutrophils.

### BTK is dispensable for neutrophil recruitment and lifespan in the Aspergillus-infected lung.

We next asked whether BTK deficiency impairs neutrophil influx or survival in the lung to increase susceptibility to pulmonary aspergillosis, since BTK has been shown to promote neutrophil recruitment in the liver, muscle, and peritoneal cavity during sterile inflammation (41, 42). However, we found a similar temporal accumulation of neutrophils in the lungs and BAL fluid of WT and *Btk^–/–^* mice during aspergillosis and observed no difference in the frequency of extravasated neutrophils in WT and *Btk^–/–^* lungs, as assessed using an intravascular staining approach (43) followed by flow cytometry–based immunophenotyping (Supplemental Figure 2, A–C). Similarly, the accumulation of other myeloid phagocytes was not decreased in the *Btk^–/–^* lungs (Supplemental Figure 2D). Further, BTK deficiency did not impair the lifespan of neutrophils and other examined myeloid phagocytes in the infected lungs (Supplemental Figure 2E). Moreover, the induction of neutrophil-targeted chemokines and of other protective proinflammatory mediators, including CXCL1, CXCL2, CCL2, IL-1β, IL-6, and TNF-α (34, 44–48), was not decreased in the *Aspergillus*-infected lungs of *Btk^–/–^* mice (Supplemental Figure 2F). Collectively, these data indicate that BTK is dispensable for neutrophil recruitment and survival at the site of infection and for the production of proinflammatory mediators in the *Aspergillus*-infected lungs.

### BTK selectively promotes neutrophil-mediated Aspergillus hyphal damage, primary granule release, and fungus-induced oxidative burst.

**Figure 2:**
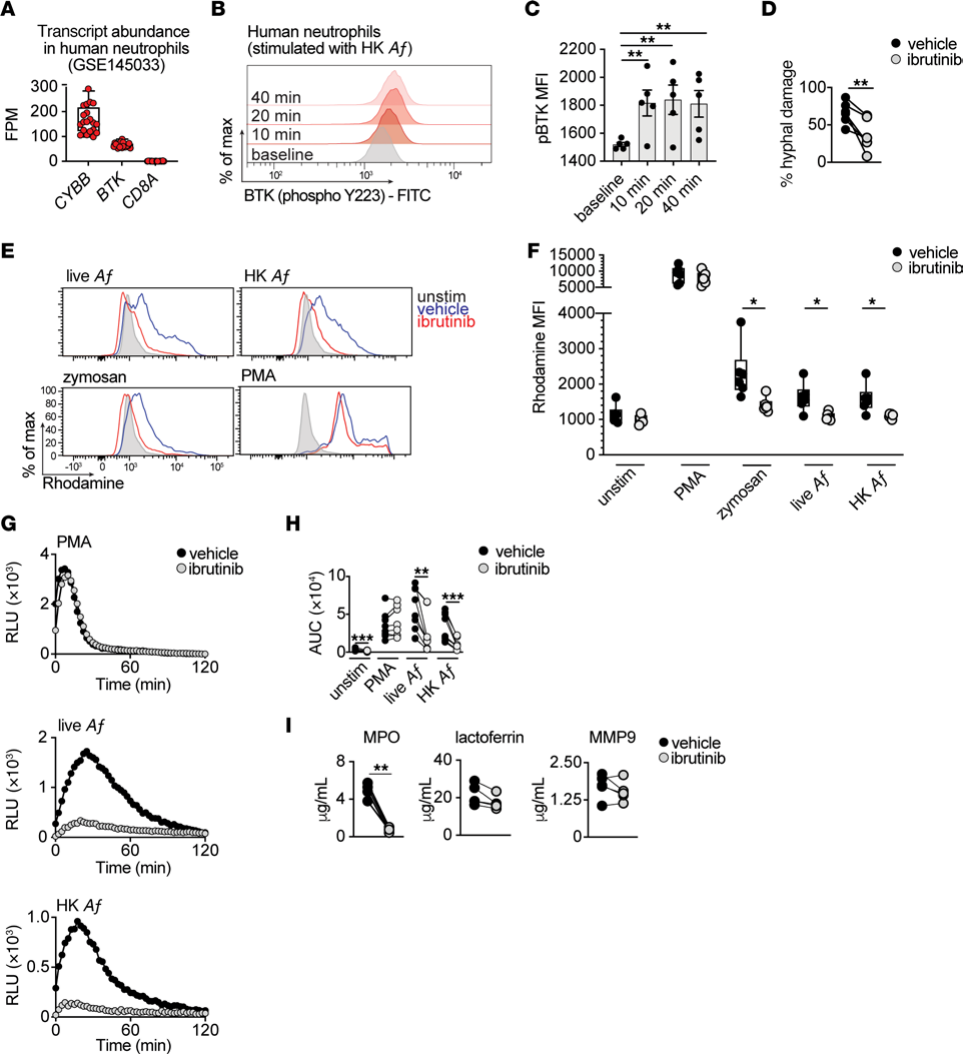
BTK is activated in human neutrophils upon fungal exposure, and pharmacologic BTK inhibition impairs antifungal effector functions. (**A**) Transcript levels of *BTK* in human neutrophils. Also shown are *CYBB* and *CD8A* as positive and negative control genes, respectively. Data sourced from GSE145033 (49, 50). (**B** and **C**) Representative FACS histograms (**B**) and geometric mean fluorescence intensity (MFI) summary data (**C**) for phosphorylated BTK in healthy donor neutrophils at baseline and at the indicated time points after stimulation with serum-opsonized, heat-killed (HK) *A*. *fumigatus* (*Af*) conidia (*n* = 5). (**D**) *A*. *fumigatus* hyphal damage induced by vehicle- or ibrutinib-treated neutrophils at an effector:target ratio of 8:1 (*n* = 6). (**E** and **F**) Representative histograms (**E**) and MFI summary data (**F**) that depict dihydrorhodamine 123 oxidation to rhodamine, in vehicle- or ibrutinib-treated healthy donor neutrophils stimulated as indicated (*n* = 6). (**G** and **H**) Luminol-amplified chemiluminescence. (**G**) Temporal trace of chemiluminescence-based reactive oxygen species (ROS) (in relative light units: RLU) of vehicle- or ibrutinib-treated healthy donor neutrophils stimulated as indicated. (**H**) AUC summary data for luminol-amplified chemiluminescence RLU shown in **G** (*n* = 9). (**I**) Vehicle- or ibrutinib-treated healthy donor neutrophils were coincubated with *A fumigatus* hyphae at an 8:1 effector:target ratio and the indicated granule components were analyzed via ELISA in the supernatants of neutrophil-hyphal cocultures (*n* = 4). Each dot represents an individual healthy donor. Quantitative data are means ± SEM (**C**). Box and whisker plots depict values ranging from minimum to maximum (**A** and **F**). Ibrutinib concentration, 250 nM. FPM, fragments per million; MPO, myeloperoxidase; MMP-9, Matrix metalloproteinase-9; PMA, phorbol-12-myristate-13-acetate; *Af*, *Aspergillus fumigatus*; HK *Af*, heat-killed *Aspergillus fumigatus*. *P<0.05, **P<0.01, ***P<0.001, determined using 1-way ANOVA with Dunnett’s multiple comparisons test (**C**), or 2-sided paired *t* test (**D**, **F**, **H**, and **I**). Grubb’s outlier test applied with 1 outlier excluded (**I**: MPO).

We then investigated whether BTK deficiency affects neutrophils qualitatively by impairing their antifungal effector functions to promote susceptibility to aspergillosis. We focused our studies primarily on human neutrophils, which effectively curtail *A*. *fumigatus* growth by phagocytosis and intracellular killing of conidial cells and via extracellular destruction of large filamentous hyphae. We sourced bulk RNA-Seq of healthy donor neutrophils (GSE145033) (49, 50) and found that they express *BTK* mRNA (Figure 2A). Upon activation, BTK is phosphorylated at Y551 by Src family kinases, leading to catalytic activation and autophosphorylation at Y223 within the BTK SH3 domain (12). Thus, we stimulated healthy donor neutrophils with serum-opsonized, heat-killed *A*. *fumigatus* conidia and observed a significant temporal induction of BTK phosphorylation (Figure 2, B and C), indicative of fungus-mediated BTK activation.

We next examined whether BTK regulates neutrophil antifungal effector functions by exposing healthy donor neutrophils to ibrutinib ex vivo at a concentration that is achievable in the plasma of ibrutinib-treated patients (4). We assessed whether ibrutinib affects the capacity of neutrophils to engulf and kill *A*. *fumigatus* conidia by using a fluorescence-based “functional microbial reporter” assay (51, 52) in which neutrophils were incubated with *A*. *fumigatus* dsRed^+^ conidia that are labeled with an Alexa Fluor 633 (AF633) tracer dye on their cell wall. Flow cytometry was used to define neutrophils that have engulfed conidia (dsRed^+^AF633^+^ and dsRed^–^AF633^+^), neutrophils that contain live (dsRed^+^AF633^+^) or killed conidia (dsRed^–^AF633^+^), and bystander neutrophils that have not engaged with conidia (dsRed^–^AF633^–^) (51, 52). Using this approach, we found that ibrutinib did not impair *A*. *fumigatus* conidial uptake and intracellular killing by neutrophils (Supplemental Figure 3, A and B) nor did it decrease neutrophil capacity to inhibit conidial germination (Supplemental Figure 3C).

By contrast, ibrutinib-mediated BTK inhibition resulted in a significant decrease in the ability of neutrophils to damage *A*. *fumigatus* hyphae (Figure 2D and Supplemental Figure 3D), which relies on their ability to produce reactive oxygen species (ROS) (38, 39). Correspondingly, patients with chronic granulomatous disease (CGD) who are defective in neutrophil ROS production due to mutations in the NADPH oxidase complex subunits are highly susceptible to aspergillosis (with a lifetime risk of approximately 40%) (2, 53, 54). Ibrutinib markedly abrogated neutrophil ROS production upon stimulation with live or heat-killed *A*. *fumigatus* conidia or zymosan particles, as assessed by dihydrorhodamine testing (Figure 2, E and F). Surprisingly, the neutrophil ROS response to phorbol-12-myristate-13-acetate (PMA), which bypasses membrane proximal activation events, was preserved (Figure 2, E and F). Similarly, temporal analysis of neutrophil ROS production by luminol-based chemiluminescence showed that ibrutinib selectively impaired ROS production upon *A*. *fumigatus*, but not PMA, stimulation (Figure 2, G and H). Neutrophil degranulation is an additional effector mechanism that contributes to the extracellular destruction of *A*. *fumigatus* hyphae (38, 39). Notably, ibrutinib markedly abrogated the release of myeloperoxidase (MPO) from neutrophil primary granules upon exposure to *A*. *fumigatus* hyphae, but it did not impair the release of lactoferrin and matrix metalloproteinase-9 (MMP-9), which reside within neutrophil secondary and tertiary granules, respectively (Figure 2I). By contrast, neutrophil extracellular traps (NETs) (55) were dispensable for *A*. *fumigatus* hyphal damage by human neutrophils (Supplemental Figure 3E), in agreement with previous observations (38, 56). Taken together, these data indicate that ibrutinib-dependent BTK inhibition selectively impairs neutrophil-dependent hyphal damage, the fungus-specific oxidative burst, and primary granule release by human neutrophils ex vivo.

**Figure 3:**
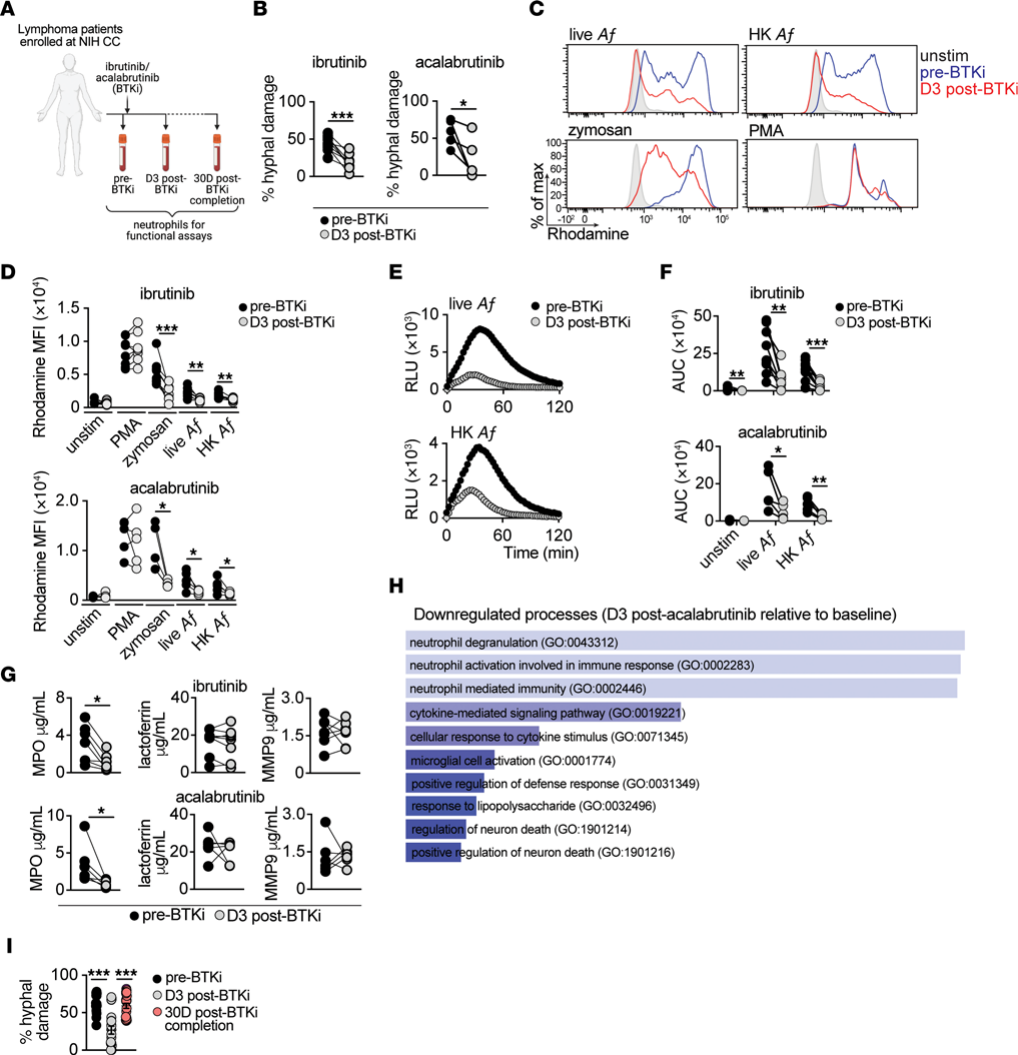
BTK inhibition in vivo, in patients with lymphoma, reduces neutrophil hyphal damage, oxidative burst, and primary granule release. (**A**) Schematic outline depicting treatment with BTKi (ibrutinib or acalabrutinib) and the timepoints of blood collection for neutrophil isolation in patients with lymphoma. (**B**–**G**) Neutrophils were isolated from patients with lymphoma before and at day 3 after treatment initiation of ibrutinib (*n* = 7-9) or acalabrutinib (*n* = 4-6). Each dot represents an individual patient. (**B**) *A*. *fumigatus* hyphal damage induced by neutrophils (effector:target ratio, 8:1) (*n* = 6). (**C** and **D**) Representative FACS histograms (**C**) and MFI summary data (**D**) depicting dihydrorhodamine 123 oxidation to rhodamine in neutrophils stimulated as indicated (ibrutinib, *n* = 8; acalabrutinib, *n* = 5). (**E** and **F**) Luminol-amplified chemiluminescence. Temporal chemiluminescence RLU trace (**E**) and AUC for RLU (**F**) of neutrophils upon stimulation as indicated (ibrutinib, *n* = 9; acalabrutinib, *n* = 4). (**G**) Patient neutrophils were coincubated with *A*. *fumigatus* hyphae (effector:target ratio, 8:1) and the indicated granule components were analyzed via ELISA in the supernatants of neutrophil-hyphal cocultures (ibrutinib, *n* = 7-8; acalabrutinib, *n* = 6). (**H**) Gene ontology (GO) terms of downregulated genes in neutrophils from patients with lymphoma isolated at day 3 after acalabrutinib treatment relative to baseline, using pseudo-bulk processing of single-cell transcriptomes (*n* = 3). (**I**) *A*. *fumigatus* hyphal damage induced by neutrophils isolated from patients with lymphoma at the indicated timepoints (effector:target ratio, 8:1). Except for panel **I**, each dot represents an individual patient. For panel **I**, data are from 3 patients, with 6 technical replicates per patient, where each dot represents a technical replicate, and quantitative data are means ± SEM. BTKi, BTK inhibitor; MPO: myeloperoxidase; MMP-9: Matrix metalloproteinase-9; PMA, phorbol-12-myristate-13-acetate; *Af*: *Aspergillus fumigatus*; HK *Af*: heat-killed *Aspergillus fumigatus*. *P<0.05, **P<0.01, ***P<0.001 using Kruskal-Wallis test with Dunn’s multiple comparisons test (**I**), or paired *t* test (**B**, **D**, **F**, and **G**).

To determine whether the defects induced by ibrutinib in healthy donor neutrophil antifungal functions ex vivo could also be observed in patients treated with BTKi in vivo*,* we compared the function of neutrophils harvested from the peripheral blood of 49 patients with lymphoma both before and at 3 days after initiation of treatment with ibrutinib or acalabrutinib (Figure 3A). Indeed, we found that a 3-day exposure to ibrutinib or acalabrutinib in vivo resulted in marked decreases in neutrophil-dependent *A*. *fumigatus* hyphal damage, in the fungus-specific oxidative burst, and in primary granule release relative to their preBTKi treatment baseline; BTKi treatment did not alter neutrophil conidial uptake (Figure 3, B–G, and Supplemental Figure 4, A and B). Consistent with these functional defects, pseudo-bulk analysis of single cell transcriptomes of patient neutrophils before and after acalabrutinib treatment identified “neutrophil degranulation”, “neutrophil activation involved in immune response”, and “neutrophil mediated immunity” gene sets curated by gene ontology (GO: 0043312, 0002283, and 0002446, respectively) as the top 3 downregulated biological processes upon acalabrutinib treatment (Figure 3H). Moreover, when we harvested neutrophils 30 days after completion of BTKi treatment, we noted restoration of the neutrophil antifungal activity to their preBTKi treatment baseline (Figure 3I), suggesting that the observed antifungal function defect arose due to BTKi therapy.

**Figure 4:**
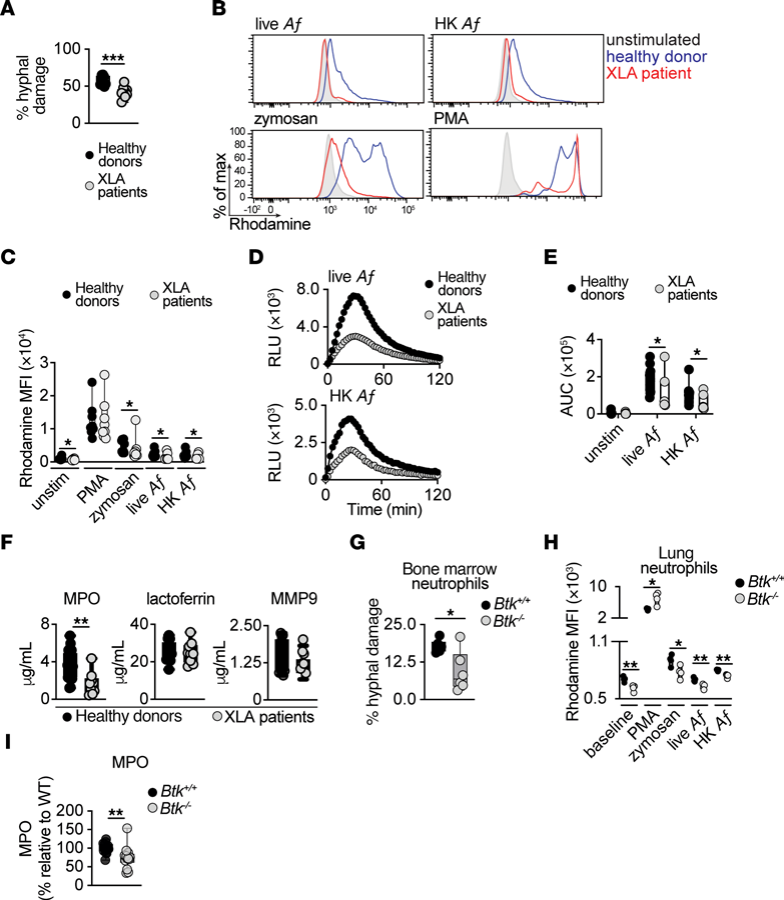
BTK promotes neutrophil hyphal damage, the oxidative burst, and primary granule release. (**A**) *A*. *fumigatus* hyphal damage induced by neutrophils from healthy donors or patients with XLA (effector:target ratio, 8:1) (*n* = 9). (**B** and **C**) Representative histograms (**B**) and MFI summary data (**C**) depicting dihydrorhodamine 123 oxidation to rhodamine in neutrophils from healthy donors or patients with XLA stimulated as indicated (*n* = 9–10). (**D** and **E**) Luminol-based assay of ROS production. Temporal trace of chemiluminescence RLU (**D**), and AUC for RLU (**E**), when neutrophils from healthy donors or patients with XLA were stimulated as indicated (*n* = 7–12). (**F**) Neutrophils of healthy donors or patients with XLA were coincubated with *A*. *fumigatus* hyphae (effector:target ratio, 8:1) and the indicated granule components were analyzed via ELISA in the supernatants (*n* = 8–22). Each dot in panels **A**, **C**, **E**, and **F** represents an individual healthy donor or a patient with XLA. (**G**) *A*. *fumigatus* hyphal damage induced by bone marrow neutrophils isolated from WT or *Btk^–/–^* mice (effector:target ratio, 32:1) (*n* = 6). (**H**) MFI summary data that depict dihydrorhodamine 123 oxidation to rhodamine in neutrophils isolated from the *Aspergillus*-infected lung of *Btk^+/+^* or *Btk^–/–^* mice at day 2 after infection, stimulated as indicated (*n* = 4). (**I**) Bone marrow neutrophils were coincubated with *A*. *fumigatus* hyphae at a 32:1 effector:target ratio and myeloperoxidase (MPO) was analyzed via ELISA in the supernatants (*n* = 6). Each dot in panels **G** and **H** represents an individual mouse, and each dot in **I** represents 1–3 technical replicates from 9 individual mice. Box and whisker plots depict values ranging from minimum to maximum (**A**, **C**, and **E**–**I**). MPO: myeloperoxidase; MMP-9: Matrix metalloproteinase-9; PMA, phorbol-12-myristate-13-acetate; Af: *Aspergillus fumigatus*; HK Af: heat-killed *Aspergillus fumigatus*; XLA: X-linked agammaglobulinemia. *P<0.05, **P<0.01, ***P<0.001 using 2 sided unpaired *t* test (**A**, **C**, and **F**–**I**), 2-sided or Mann-Whitney *U*-test (**C** and **E**).

To specifically examine the direct role of BTK deficiency on neutrophil anti-*Aspergillus* functions, we analyzed neutrophils from 9 patients with XLA who harbor germline loss-of-function *BTK* variants (57). Similar to the defects observed in neutrophils upon ex vivo and in vivo exposure to ibrutinib or acalabrutinib, neutrophils from patients with XLA exhibited selective impairments in *A*. *fumigatus* hyphal damage, fungus-induced ROS production, and primary granule release, whereas conidial uptake and intracellular killing capacity, PMA-induced ROS production, and secondary and tertiary granule release were intact (Figure 4, A–F, and Supplemental Figure 4, C and D). In addition, neutrophils from *Btk^–/–^* mice also exhibited impaired *A*. *fumigatus* hyphal killing, fungus-specific oxidative burst, and MPO degranulation (Figure 4, G–I); by contrast, conidial uptake and intracellular killing were unaffected, which we confirmed in *S100a8-Cre/Btk^fl/fl^* neutrophils (Supplemental Figure 4, E and F). Collectively, these data demonstrate an essential role for BTK in regulating selective *Aspergillus*-directed protective functions in human and murine neutrophils.

### BTK promotes the activation of p40^phox^ and RAC2 in human neutrophils.

**Figure 5:**
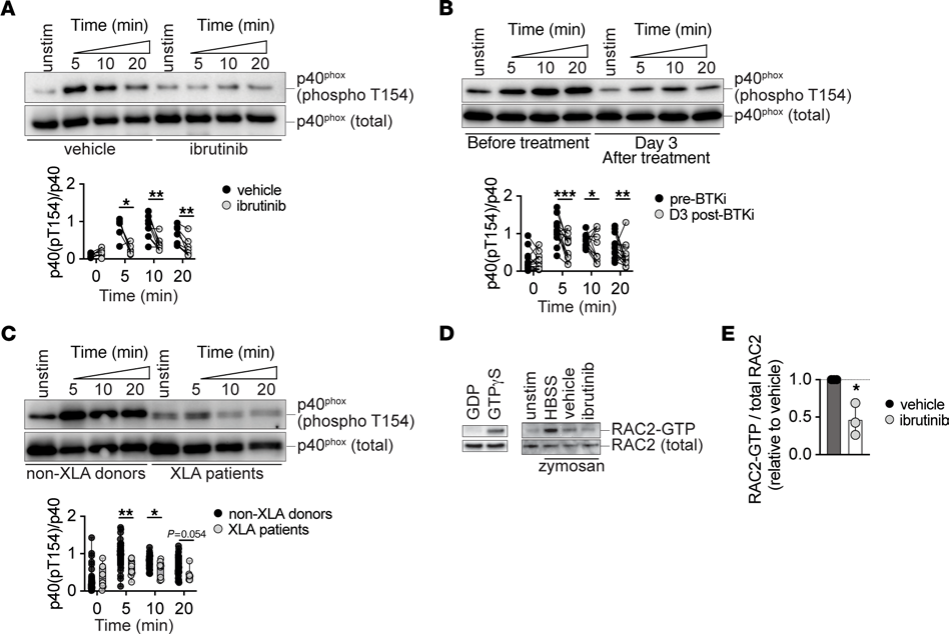
BTK mediates p40^phox^ and RAC2 activation in human neutrophils. (**A**–**C**) Immunoblot analysis of p40^phox^ phosphorylation (at T154) in human neutrophils upon stimulation with serum-opsonized heat-killed *Aspergillus* conidia at the indicated time points. Representative immunoblot images (top panels) and quantified pixel density values (lower panels) are shown. p40^phox^ phosphorylation is shown in healthy donor neutrophils treated with vehicle or ibrutinib (**A**), in neutrophils from patients with lymphoma, isolated before or at day 3 after initiation of treatment with ibrutinib or acalabrutinib (**B**), and in neutrophils isolated from healthy donors or patients with XLA (**C**). (**D**) Representative immunoblot images depicting active RAC2-GTP and total RAC2. Left: pull-down was performed using unstimulated healthy donor neutrophil lysates in the presence of GDP (negative control) and GTPγS (positive control). Right: pull-down was performed using healthy donor neutrophil lysates, following neutrophil treatment with vehicle, ibrutinib, or buffer (Hank’s balanced salt solution: HBSS) and stimulation with serum-opsonized zymosan. (**E**) Quantification of the ratio of active RAC2-GTP relative to total RAC2, normalized to the vehicle-treated neutrophils. Each dot depicts an individual healthy donor or patient. Box and whisker plots depict values ranging from minimum to maximum (**C**). Bars depict mean ± SD. Ibrutinib concentration, 2.5 μM. BTKi, BTK inhibitor. *P<0.05, **P<0.01, ***P<0.001, determined using 2-sided paired *t* test (**A** and **B**), 2-sided Wilcoxon test (**B**), 2 sided unpaired *t* test (**C**) or 2-sided Mann-Whitney *U* test (**C**), or 2-sided Welch’s *t* test (**E**). Grubb’s outlier test applied with 1 outlier excluded (**C**: 20 min timepoint).

We next aimed to gain mechanistic insights into how BTK inhibition adversely affects the neutrophil oxidative burst and granule release. Our finding that BTK deficiency impairs neutrophil ROS production upon fungal cell or fungal particulate stimulation while the PMA-induced oxidative burst is preserved is reminiscent of a similar selective defect reported in patients with a subtype of CGD. This subtype of CGD is caused by genetic deficiency of *NCF4*, which encodes p40^phox^, the fifth NADPH oxidase complex subunit, and patients with this deficiency do not lose PMA-induced ROS production (58, 59). By contrast, neutrophils from patients with CGD caused by genetic deficiency of any of the other NADPH oxidase complex subunits that encode gp91^phox^, p47^phox^, p67^phox^, and p22^phox^ or of the transmembrane membrane chaperone, *EROS*, exhibit loss of PMA-induced ROS production (60–62). Hence, we hypothesized that BTK regulates p40^phox^ activity. Because p40^phox^ phosphorylation at the conserved T154 residue is required for activation of its oxidase activity (63), we assessed whether BTK inhibition affects p40^phox^ phosphorylation at T154. We stimulated healthy donor neutrophils with heat-killed *A*. *fumigatus* conidia ex vivo and found a decreased temporal induction of p40^phox^ phosphorylation in the presence of ibrutinib (Figure 5A) or acalabrutinib (Supplemental Figure 5). A similar defect in *Aspergillus*-mediated p40^phox^ phosphorylation induction was also observed in neutrophils harvested from patients with lymphoma 3 days after initiation of ibrutinib treatment (Figure 5B) and in neutrophils from patients with XLA (Figure 5C). Thus, BTK-dependent neutrophil ROS production upon fungal-specific stimulation is associated with BTK-mediated activation of the NADPH oxidase complex subunit p40^phox^.

Considering the selective defect in release of primary — but not secondary or tertiary — granules by neutrophils in the setting of BTK inhibition, we focused on the Rho-family guanosine triphosphatase (GTPase) RAC2, which is an essential mediator of neutrophil primary — but not secondary or tertiary — granule release in response to diverse stimuli, including N-formyl-Met-Leu-Phe (64–66). Thus, we hypothesized that BTK promotes primary granule release by activating RAC2. RAC2 activation entails the release of inactive, GDP-bound RAC2 from the guanine nucleotide dissociation inhibitor, RhoGDI, followed by its association with a guanine exchange factor (GEF), such as TIAM1 (67). The interaction of RAC2 with the GEF releases GDP and promotes the binding of GTP to RAC2, thereby promoting RAC2 activation. In turn, RAC2-GTP binds to and activates several downstream effector molecules such as p21-activated kinase 1 (PAK1) to drive cellular functions such as ROS production (68, 69). To test whether BTK inhibition affects RAC2 activation, we stimulated healthy donor neutrophils with zymosan particles ex vivo in the presence or absence of ibrutinib. We lysed neutrophils and precipitated proteins bound to the PAK1 protein binding domain (PBD) that was fused to glutathione-*S*-transferase and immobilized on glutathione agarose. By performing immunoblot analysis for RAC2 and normalizing for total RAC2 content, we found decreased levels of PAK1-PBD-associated RAC2 in zymosan-stimulated neutrophils that were exposed to ibrutinib relative to ibrutinib-unexposed neutrophils (Figure 5, D and E). This finding indicates that significantly less RAC2 is present in its active GTP-bound form in the setting of BTK inhibition. Taken together, these data support the notion that BTK regulates p40^phox^ and RAC2 activation to promote the fungus-elicited neutrophil oxidative burst and primary granule release.

### BTK is activated downstream of TLR2, FcγR, and Dectin-1 in human neutrophils.

**Figure 6:**
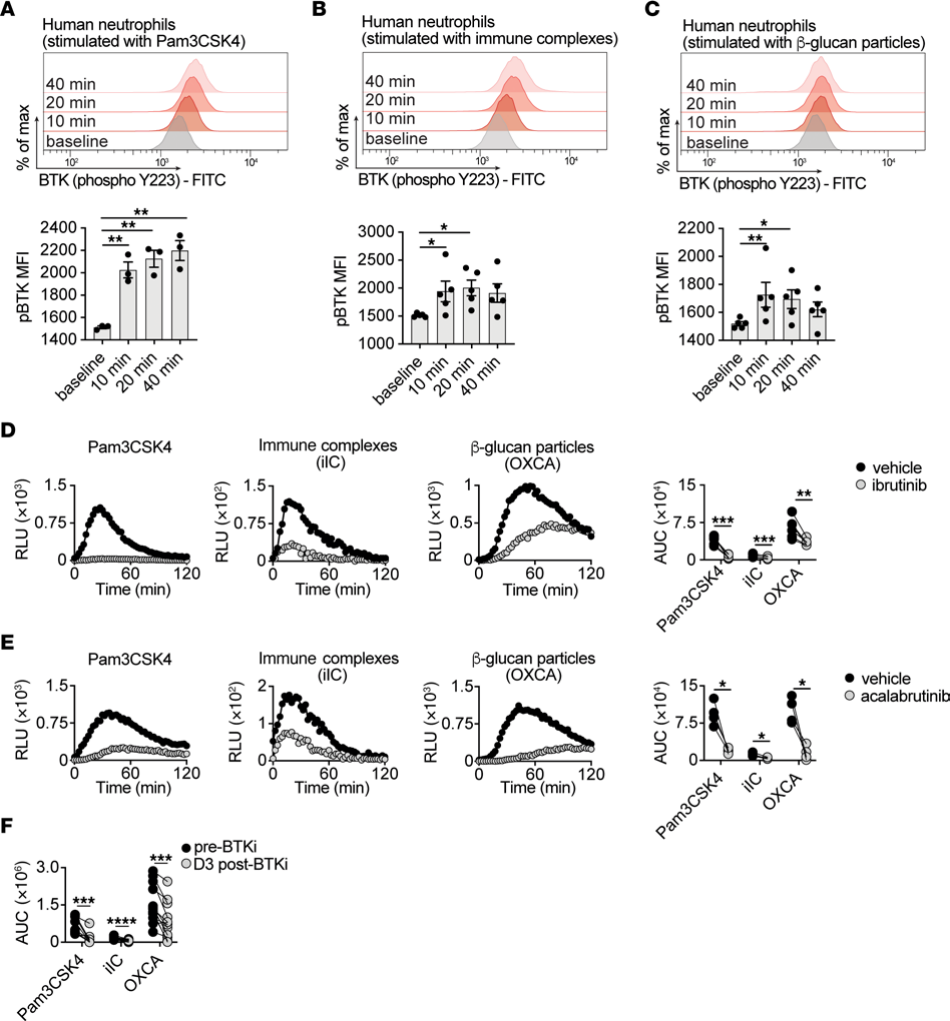
BTK acts downstream of TLR2, FcγR, and Dectin-1 engagement to promote the neutrophil oxidative burst. (**A**–**C**) Representative FACS histograms (upper panels) and mean fluorescence intensity (MFI) summary data (lower panels) for phosphorylated BTK (at Y223) in healthy donor neutrophils at baseline and at the indicated timepoints after stimulation with the TLR2 agonist Pam3CSK4 (**A**), FcγR-engaging immobilized immune complexes (iIC) (**B**) or Dectin-1-engaging β-glucan particles (OXCA) (**C**) (*n* = 5). (**D** and **E**) Luminol-based assay of reactive oxygen species (ROS) production in human neutrophils. Representative temporal traces of chemiluminescence (left panels) and AUC for luminol-amplified chemiluminescence relative light units (RLU) (right panels) when vehicle or ibrutinib-treated (**D**) or acalabrutinib-treated (**E**) healthy donor neutrophils were stimulated as indicated (*n* = 4–6). (**F**) AUC of luminol-amplified chemiluminescence RLU in neutrophils isolated from ibrutinib- or acalabrutinib- treated lymphoma patients, before and at day 3 after treatment initiation and stimulated as indicated (*n* = 12). Each dot represents an individual healthy donor or patient. Quantitative data are means ± SEM (**A**–**C**). Ibrutinib concentration, 250 nM. BTKi, BTK inhibitor; Pam3CSK4: Pam3CysSerLys4; iIC: immobilized immune complexes; OXCA: β-glucan particles (NaCLO-oxidized *Candida albicans*). *P<0.05, **P<0.01, ***P<0.001, ****P<0.0001 using repeated measures 1-way ANOVA with Šidák’s multiple comparisons test (**A**–**C**), or 2-sided paired *t* test (**D**–**F**) or 2-sided Wilcoxon test (**F**).

We next aimed to define the fungal-sensing cell surface receptors that act upstream of BTK to drive its activation and to promote BTK-dependent function in human neutrophils. In addition to BTK activation following stimulation with heat-killed *A*. *fumigatus* serum-opsonized conidia (Figure 2, B and C), we also observed induction of BTK phosphorylation in healthy donor neutrophils upon exposure to zymosan particles by using a human whole blood stimulation model (70) (Supplemental Figure 6A). Zymosan signals through Toll-like receptor 2 (TLR2) and the C-type lectin receptor, Dectin-1 (71). Ligation and signaling through the immunoglobulin Fc receptor (FcγR) is likely also involved in this experimental setting considering the high levels of opsonization within whole blood. Therefore, to determine which of these receptors may directly activate BTK, we assessed BTK phosphorylation in healthy donor neutrophils after stimulation with specific ligands for TLR2 (Pam3CSK4), FcγRIIA/IIIB (immobilized immune complexes) (72), and Dectin-1 (β-glucan particles) (73). Engagement of each of these 3 receptors led to an increased temporal induction of BTK phosphorylation (Figure 6, A–C). Moreover, *Aspergillus*-induced BTK phosphorylation was abrogated in the presence of pharmacological inhibitors directed against TLR2, FcγRIIA/IIIB, or Dectin-1 (Supplemental Figure 6B). These observations point to TLR2-, FcγR-, and Dectin-1-mediated activation of BTK-dependent signaling in human neutrophils. We then examined whether engagement of any of these receptors promotes ROS production in human neutrophils and found that ligation of TLR2, FcγR, or Dectin-1 (Figure 6, D and E), but not of TLR4 or TLR9 (data not shown), did so. Engagement of TLR2, but not FcγR or Dectin-1, also augmented the capacity of human neutrophils to damage *Aspergillus* hyphae (Supplemental Figure 6C). Notably, ibrutinib- or acalabrutinib-induced BTK inhibition significantly reduced TLR2-, FcγR-, and Dectin-1-mediated ROS production in healthy donor neutrophils ex vivo (Figure 6, D and E). Similarly, neutrophils harvested from lymphoma patients 3 days after initiation of treatment with ibrutinib or acalabrutinib displayed defective TLR2-, FcγR-, and Dectin-1-mediated ROS production relative to their preBTKi treatment baseline (Figure 6F). Collectively, these data show that TLR2, FcγRIIA/IIIB, and Dectin-1 act as upstream activators of BTK signaling to mediate anti-*Aspergillus* function in human neutrophils.

### GM-CSF partially rescues the neutrophil functional defects caused by BTK inhibition and improves the survival of Aspergillus-infected Btk^–/–^ mice.

Next, we wondered whether we could employ a translational immune modulatory approach to bypass the observed BTKi-driven impairment in anti-*Aspergillus* neutrophil function for potential therapeutic benefit. We focused on IFN-γ, which is FDA-approved to decrease the frequency and severity of infections in CGD (74), granulocyte colony-stimulating factor (G-CSF) and granulocyte-macrophage–colony stimulating factor (GM-CSF), which are FDA-approved to accelerate myeloid reconstitution following hematopoietic stem cell transplantation or myeloablative chemotherapy (75). All 3 cytokines prime various neutrophil functions, including potentiating oxidative burst to subsequent stimuli (76–81).

**Figure 7:**
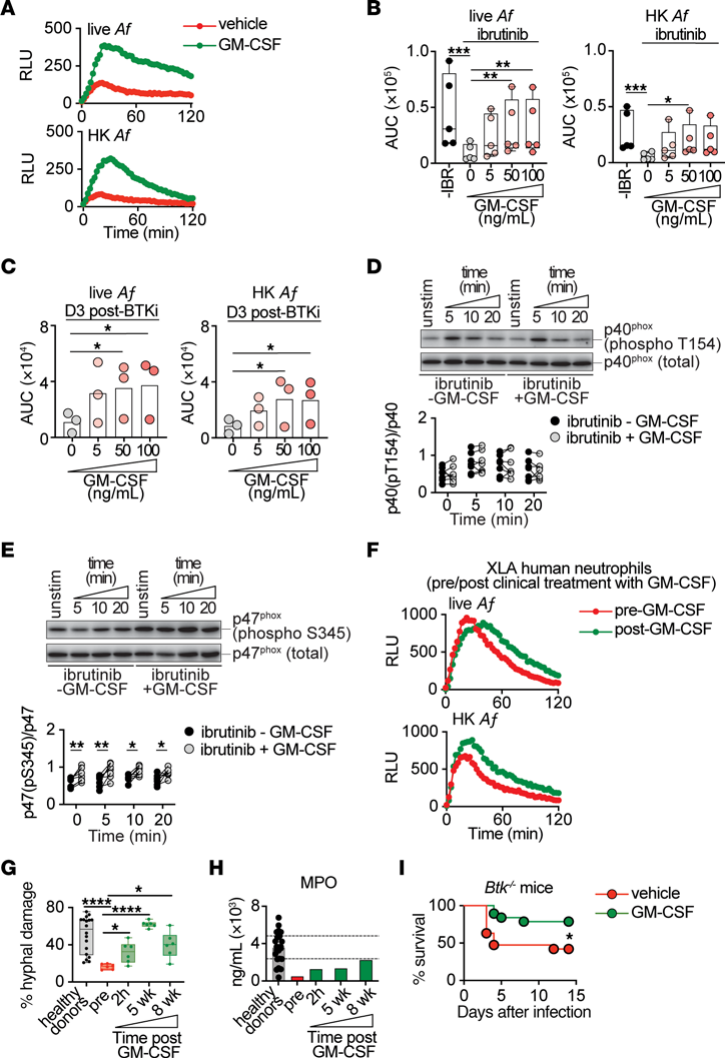
GM-CSF rescues BTK-inhibited neutrophil functional defects and improves survival in *Aspergillus*-infected *Btk^–/–^* mice. (**A**–**C**) Luminol-amplified chemiluminescence. Representative relative light units (RLU) trace of ibrutinib-treated healthy donor neutrophils (**A**), and AUC for RLU in vehicle- or ibrutinib-treated healthy donor (**B**) or BTKi-treated lymphoma patient neutrophils (**C**), in response to 50 ng/mL (**A**) or increasing concentrations of GM-CSF (**B** and **C**). Each dot depicts an individual donor (**B**, *n* = 5; **C**, *n* = 3). (**D** and **E**) Representative immunoblot images (top) and pixel density (bottom) of p40^phox^ (phospho-T154; **D**) and p47^phox^ (phospho-S345; **E**) upon GM-CSF (50 ng/mL) treatment, in ibrutinib-treated, HK *Af* stimulated healthy donor neutrophils. (*n* = 7). (**F**–**H**) Neutrophils were isolated from a patient with XLA, at baseline, 2 hours, 5 weeks, and 8 weeks after GM-CSF treatment (500 μg, Sargramostim injection, 3× weekly). (**F**) Representative RLU trace at baseline and 2 hours after GM-CSF treatment initiation, upon stimulation as indicated. (**G** and **H**) *A*. *fumigatus* hyphal damage (**G**) and MPO release upon hyphal coincubation (**H**) (effector:target ratio, 8:1). Each dot represents a single donor (healthy donors; **G** and **H**), or a technical replicate (XLA patient; **G**). (**I**) Survival of GM-CSF or vehicle-treated *Btk^–/–^* mice after infection with *A*. *fumigatus* (*n* = 20). Ibrutinib concentration, 250 nM (**A**–**C**) or 2.5 μM (**D** and **E**). BTKi, BTK inhibitor; IBR, ibrutinib; XLA: X-linked agammaglobulinemia; GM-CSF: granulocyte-macrophage colony-stimulating factor; *Af*: *Aspergillus fumigatus*; HK *Af*: heat-killed *Aspergillus fumigatus*. Bars depict mean values (**C** and **H**). Box and whisker plots depict range from minimum to maximum (**B** and **G**). Dotted lines mark 25th and 75th percentile MPO values for healthy donor neutrophils (**H**). *P<0.05, **P<0.01, ***P<0.001, ****P<0.0001, determined using repeated measures 1-way ANOVA with Dunnett’s multiple comparisons test (**B**, *live-Af*; **C**), Friedman’s test with Dunn’s multiple comparison test (**B**, *HK-Af*), 2-sided paired *t* test (**D**, **E**, and **G**), Mann-Whitney *U* test (**G**), or log-rank test (**I**).

Although IFN-γ and G-CSF did not improve *Aspergillus*-induced ROS production in ibrutinib-exposed neutrophils (Supplemental Figure 7, A and B), we observed a GM-CSF–induced increase in *Aspergillus*-induced ROS production in ibrutinib-treated healthy donor neutrophils, which was also evident in neutrophils not exposed to ibrutinib (Figure 7, A and B, and Supplemental Figure 7C). We observed a similar priming of *Aspergillus*-induced ROS production with GM-CSF treatment ex vivo in neutrophils harvested from patients with lymphoma 3 days after treatment initiation with ibrutinib or acalabrutinib (Figure 7C). The GM-CSF-mediated boosting in oxidative burst was selective, as it was not observed in ibrutinib-exposed neutrophils following PMA stimulation (Supplemental Figure 7D). We next wondered whether GM-CSF increased ROS production in ibrutinib-exposed neutrophils by correcting the BTKi-driven defect in p40^phox^ activation or by bypassing it through phosphorylation of the p47^phox^ subunit at S345, as previously described (80, 81). GM-CSF treatment of ibrutinib-treated healthy donor neutrophils ex vivo led to enhanced levels of p47^phox^ phosphorylation at S345, without affecting p40^phox^ phosphorylation (Figure 7, D and E). Thus, GM-CSF boosts ROS production in BTKi-exposed neutrophils through enhanced p47^phox^ activation.

To contextualize the potential impact of GM-CSF-mediated priming of neutrophil function in the setting of BTK deficiency in vivo, we harvested neutrophils from a patient with XLA before and at different time-points after initiation of GM-CSF treatment. We noted an improvement in *Aspergillus*-induced ROS production, *A*. *fumigatus* hyphal damage, and MPO release in XLA neutrophils harvested post-GM-CSF treatment relative to their pretreatment baseline (Figure 7, F–H). Moreover, treatment of *Btk^–/–^* mice with GM-CSF conferred a significant survival benefit after aspergillosis relative to *Btk^–/–^* mice that did not receive GM-CSF (survival, 79% versus 42%, respectively) (Figure 7I). Taken together, these data demonstrate the beneficial effects of GM-CSF on neutrophil antifungal function in the setting of BTK deficiency and inform a potential translational strategy to bypass the BTKi-driven neutrophil defects during aspergillosis.

## Discussion

Herein, we uncover the crucial role of BTK-dependent neutrophil activation in defense against systemic fungal infection. We show that BTK becomes activated in neutrophils following engagement of TLR2, FcγR, and Dectin-1, leading to induction of fungus-specific ROS production and to primary granule release. Mechanistically, BTK facilitates the activation of the NADPH oxidase complex subunit p40^phox^ and the GTPase RAC2 to promote damage to the tissue-invasive hyphal form of *Aspergillus*. Importantly, GM-CSF partially restores these deficits by activating p47^phox^, suggesting a potential therapeutic intervention for patients who have been treated with BTKi. Our conclusions (illustrated in Supplemental Figure 8) are based on comprehensive analyses of immunological, functional, transcriptional, and biochemical parameters from diverse human cohorts, including healthy donors, patients who have been treated with BTKi and patients with XLA, and corroborating investigations in WT and *Btk^–/–^* mice. Our study identifies BTK as a critical mediator of neutrophil antimicrobial function, elucidates the mechanisms underlying the susceptibility to fungal disease of patients who have been treated with BTKi, and provides the foundation for developing a GM-CSF–based translational strategy to bypass BTKi-driven neutrophil impairment.

Our study aimed to investigate the mechanisms underlying the unexpected development of aspergillosis in patients undergoing BTKi treatment. While BTK is well-known for its involvement in B cell function (11, 12), B cells are dispensable for *Aspergillus* immune surveillance (2). Thus, we focused our studies on the role of BTK in neutrophils, as these cells are the principal effectors against *Aspergillus* (2) and because we found that *Btk*-deficient mice, as well as mice with myeloid phagocyte-specific and neutrophil-specific *Btk* ablation, were susceptible to aspergillosis, indicating that BTK plays a critical role in promoting neutrophil-mediated defense against this fungal infection. Future studies will be needed to examine whether BTK-driven activation of monocytes/macrophages may also contribute to anti-*Aspergillus* defense, especially at the levels of early conidial killing and inhibition of conidial germination. It is possible that BTK inhibition may impair conidial killing by resident macrophages in the lung, as suggested for human monocyte-derived macrophages and monocyte-differentiated nurse-like cells, thereby enabling conidial germination into hyphae, which cannot be effectively controlled by BTK-inhibited neutrophils (24, 26, 27, 31, 35, 82). Moreover, because aspergillosis in the setting of BTKi treatment has a high predilection for extrapulmonary spread, particularly to the central nervous system (4, 83), the role of BTK in promoting microglial anti-*Aspergillus* responses merits further investigation.

Our data show that TLR2, FcγR, and Dectin-1, but not TLR4 or TLR9, are the fungal-sensing surface receptors that act upstream to activate BTK, triggering neutrophil antifungal function. By contrast, prior work in murine macrophages suggested that phagosomal TLR9 is involved in promoting BTK-dependent NFAT activation upon *A*. *fumigatus* exposure (27), though fungus strain-specific attributes may account for observed differences in receptor engagement. Moreover, we demonstrated that BTK inhibition selectively impairs certain neutrophil effector functions, such as extracellular hyphal damage, the fungus-induced oxidative burst, and primary granule release, whereas other functions such as conidial uptake and intracellular killing, PMA-induced ROS production, and the release of secondary and tertiary granules remained intact. These observations align with previous studies revealing different mechanisms employed by human neutrophils for the clearance of *Aspergillus* conidia versus hyphae. As such, neutrophils rely on lactoferrin-dependent iron sequestration for effective conidial killing in the absence of NADPH oxidase-dependent ROS, whereas MPO release and ROS production, but not lactoferrin release, are essential for hyphal killing (38, 84). In agreement, we show that BTKi-driven neutrophil defects in ROS production and MPO release — while lactoferrin release remains intact — impair hyphal, but not conidial, killing. Future studies are warranted to dissect the relative roles of BTK-dependent intracellular versus extracellular ROS production in hyphal killing and to examine whether BTK also promotes neutrophil antifungal responses against *A*. *nidulans*, a species that characteristically affects patients with CGD (85). The dispensable role of BTK in *Aspergillus* conidial uptake by neutrophils expands upon a similar finding in murine macrophages (27) but contrasts with the reported BTK dependence for phagocytosis of *Candida albicans* and zymosan particles by murine macrophages and CD14^+^ human monocytes, underscoring phagocyte-specific and fungus-specific effects of BTK in this context (26, 86). Importantly, these selective neutrophil deficits were evident not only during BTKi treatment in healthy donor neutrophils exposed to BTKi ex vivo and in neutrophils harvested from patients with lymphoma who underwent BTKi treatment, but also in neutrophils from patients with XLA who have nonfunctional BTK due to inherited *BTK* deficiency, and in *Btk^–/–^* mouse neutrophils. These results firmly establish the specific dependence on BTK for these neutrophil functions. Further, these results emphasize the importance of examining whether neutrophil dysfunction, besides antibody deficiency, contributes to the increased susceptibility to bacterial infections in BTKi-treated and XLA patients (5, 17).

The specific defects observed in fungus-induced ROS production and primary granule release, which are processes known to be selectively governed by p40^phox^ and RAC2, respectively (58, 59, 64–66), prompted investigations that uncovered the detrimental role of BTK inhibition in the activation of p40^phox^ and RAC2 in human neutrophils, revealing the molecular mechanisms of BTKi-driven aspergillosis susceptibility. Future studies will be needed to further characterize the biochemical basis of BTK-dependent p40^phox^ and RAC2 activation in neutrophils by defining the BTK intracellular partners involved in these processes.

It is important to note that aspergillosis has not been observed in the limited number of patients reported with inherited p40^phox^ and RAC2 deficiencies (58, 87–89). Evaluation of a larger number of such patients will provide a more comprehensive understanding of their predisposition, or lack thereof, to aspergillosis. The increased risk of aspergillosis in patients who have been treated with BTKi is likely a result of the combined defects in p40^phox^ and RAC2 activation caused by BTKi treatment, which may synergistically heighten susceptibility relative to each defect alone. Moreover, patients who have been treated with BTKi only experience neutrophil defects due to BTK inhibition, but they may also exhibit additional immunological impairments arising from their underlying hematological disorder and/or advanced age (90, 91). These additional factors of predisposition potentially further enhance the risk of aspergillosis and likely contribute to the higher incidence of aspergillosis in this group (4–10) relative to the rare reports of aspergillosis in patients with XLA (22, 23). Interestingly, a similar differential susceptibility to fungal infection between acute pharmacological inhibition of an immunological pathway and inherited deficiency of the same pathway has also been observed with C5-targeted monoclonal antibody therapy and inherited *C5* deficiency (92–94). These observations support the concept that acute pharmacological inhibition may not allow for the timely development of compensatory mechanisms, unlike the scenario of lifelong inherited deficiency of the same pathway. In summary, the greater predisposition to aspergillosis in patients who have been treated with BTKi relative to patients with XLA is likely multifactorial, resulting from a combination of BTKi-driven neutrophil deficits, additional immunological impairments caused by underlying conditions, chemotherapy, corticosteroid administration, or age, and potential differences between pharmacologic inhibition and inherited deficiency of immunological pathways. Possible off-target effects of BTKi on other protective immune factors cannot be excluded. These findings collectively underscore the importance of vigilant surveillance for fungal and other opportunistic infections in patients receiving immune pathway–modulating biologics, even when such infections may not have been frequently observed in inborn errors of immunity that affect the same pathway.

An important translational finding from our research is that the FDA-approved cytokine GM-CSF, but not G-CSF or IFN-γ, restored the defects in neutrophil function caused by BTK inhibition both in vitro and in vivo. This improvement was achieved through enhanced p47^phox^ activation. Of note, a companion report submitted by Vargas-Blanco and colleagues found that TNF-α rescued BTKi-driven neutrophil deficits**)** (95). TNF-α was previously shown to prime neutrophil ROS production through enhanced p47^phox^ activation (81), similar to GM-CSF, suggesting that the molecular mechanism of bypassing BTKi-driven neutrophil defects may converge at the level of p47^phox^ phosphorylation at S345. We recently showed that GM-CSF restored antifungal immune defects in the gut and improved host survival in a mouse model of antibiotic-induced invasive candidiasis (96). In addition, GM-CSF signaling is essential for host defense and neutrophil antifungal activity in a murine pulmonary *A*. *fumigatus* infection model, and administration of GM-CSF accelerates *Aspergillus* clearance in WT mice (97). Moreover, a Phase IV randomized clinical trial revealed that GM-CSF reduced mortality associated with invasive fungal infections in recipients of hematopoietic stem cell transplants (98). Our data suggest that GM-CSF may be a promising adjunct immunotherapeutic approach to counteract neutrophil deficits and improve fungal infection outcomes in patients receiving BTKi treatment. These results warrant further investigation in a clinical trial setting to assess the efficacy and safety of GM-CSF in this context.

Overall, our work provides mechanistic and translational insights into how BTK orchestrates neutrophil-dependent defense during fungal infection, thereby illuminating the mechanisms underlying increased susceptibility to fungal disease of patients who have been treated with BTKi.

## Methods 

### Sex as a biological variable

Our study utilized both men and women as human donors and male and female animals. Similar findings were observed irrespective of sex.

### Mice

*Btk^–/–^* and *Btk^fl/fl^* mice were provided by Wasif Khan and Emily Clark (University of Miami, Coral Gables, Florida, USA) (11, 99). C57BL/6J mice (JAX strain 000664), and *Rag2^–/–^* mice (JAX strain 008449) (100) were purchased from the Jackson laboratory. *Lyz2-Cre^tg^* mice (101) and *S100a8-Cre^tg^* mice (102) were crossed with *Btk^fl/fl^* mice (99) to generate *Lyz2-Cre/Btk^fl/fl^* and *S100a8-Cre/Btk^fl/fl^* lines, respectively. Mice aged 8–14 weeks were used and procedures were performed according to guidelines set forth by the Guide for the Care and Use of Laboratory Animals under the auspices of approved protocols by the NIAID (LCIM14E) and Memorial Sloan Kettering Cancer Center (no. 13-07-008) Animal Care and Use Committees. 

### Participants

#### Healthy donors.

Healthy donors (age, 23–82 years old; 92 male samples, 43 female samples) were enrolled in protocols approved by the NIH Institutional Board Review between 2017 and 2023 (for information on healthy donor eligibility criteria please refer to identifiers NCT00001846 and NCT01386437 on ClinicalTrials.gov). The donors provided written informed consent and were deidentified before the research blood was distributed for downstream processing. Specifically, 20 mL sodium heparin or EDTA-treated blood was collected and utilized for downstream analyses as described below. For analyzing phosphorylated BTK levels in neutrophils after inhibition of pattern recognition receptors, buffy coats from deidentified healthy donors (*n* = 5) were purchased from the New York Blood Center. The buffy coats were utilized within 24 hours of collection.

#### Patients with lymphoma treated with ibrutinib or acalabrutinib.

The TEDDI-R study in which ibrutinib was administered with temozolomide, etoposide, doxorubicin, dexamethasone, and rituximab was approved by the NIH Institutional Review Board (IRB) and patients provided written informed consent (ClinicalTrials.gov, NCT02203526 and NCT03964090). The patients’ demographic characteristics, clinical information, and outline of research evaluations are outlined in Supplemental Table 1. Eligible patients had primary CNS lymphoma (PCNSL) diagnosis (ClinicalTrials.gov, NCT02203526) or aggressive B cell lymphomas with secondary involvement of the CNS (sCNSL), untreated or relapsed/refractory disease, or untreated B cell lymphoma with CNS involvement (ClinicalTrials.gov, NCT03964090). In the acalabrutinib study, participants with untreated diffuse large B cell lymphoma were enrolled on a NIH IRB approved protocol (ClinicalTrials.gov, NCT04002947), in which oral acalabrutinib was initially administered twice daily for 14 days. Thereafter, patients were treated with DA-EPOCH-R or R-CHOP therapy with or without acalabrutinib continuation, depending on their initial tumor response to acalabrutinib monotherapy. For the purpose of our study, 20 mL sodium-heparin or EDTA-treated blood was drawn from patients 1 day prior and 3 days after ibrutinib or acalabrutinib monotherapy administration. For some patients, blood was also collected 30 days after BTKi treatment completion. Neutrophils from a total of 49 patients with lymphoma (age, 34-85 years old; 39 male, 10 female) were evaluated in our study between 2018 and 2022 (Supplemental Table 1).

#### Patients with XLA.

Nine patients with XLA who were male with ages ranging from 18 to 56 years were enrolled on protocols approved by the NIH Institutional Board Review (ClinicalTrials.gov, NCT00001244, NCT00001355) between 2018 and 2022. The patients provided written informed consent and were deidentified before the research blood was distributed for downstream processing. The patients’ demographic characteristics, *BTK* mutation information, and outline of research evaluations are summarized in Supplemental Table 2. Sodium-heparin–treated blood or EDTA-treated blood was utilized, and primary human neutrophils were isolated for downstream assays (see section “Isolation of primary human neutrophils” below). In addition, non-XLA healthy donor blood was also collected on the same day as control for functional analyses, as described under the “Healthy donors” section above.

### Isolation of primary human neutrophils and analysis of anti-Aspergillus effector functions

Blood collected from the participants, as described above, was diluted with PBS (Corning, 21-040-CM) and lymphocyte separation media (LSM) (Corning, 25-072-CI) and was used to separate leukocytes after spinning at 800*g* with acceleration of 5 and break of 3 for 25 minutes. After removal of the peripheral blood mononuclear cells (PBMCs) from the interphase, the remaining neutrophils and RBCs were gently mixed with 3% dextran solution (SERVA, 18696.01) in 0.85% NaCl (J.T. Baker, 3624-01) to allow for separation of neutrophils from the RBCs. Remaining RBCs were osmotically lysed using 0.2% NaCl followed by resuspension in 1.6% NaCl. The neutrophils were then resuspended in PBS, counted, and adjusted to appropriate concentrations for downstream effector function assays to probe fungal uptake, intracellular killing, conidial germination inhibition, hyphal damage, degranulation, and oxidative burst; detailed methods describing the effector functions assays are provided in Supplemental Methods.

For RAC2 activation assay, single-cell RNA-Seq, and flow cytometry–based assay to probe for BTK phosphorylation, human neutrophils were isolated via negative immunomagnetic separation, which was performed on EDTA-treated whole blood or 1mM EDTA-supplemented buffy coats using the EasySep Direct Human Neutrophil Isolation Kit (STEMCELL Technologies, 19666), as per the manufacturer’s instructions; detailed procedures for flow cytometry–based BTK phosphorylation assay, RAC2 activation assay, and single-cell RNA-Seq are provided in Supplemental Methods.

### Evaluation of NADPH oxidase subunit phosphorylation in human neutrophils

Neutrophils were stimulated and NADPH oxidase subunit activation was analyzed as previously described by El-Benna et al. (103) and detailed in Supplemental Methods.

### Mouse pulmonary Aspergillus infection 

Mice were anesthetized using isoflurane, and the pharyngeal aspiration technique was performed for *Aspergillus* inoculation as described previously (104). Briefly, mice were inclined with tongue extended using forceps, whereafter 50 μL of *A*. *fumigatus* conidial suspension, prepared as described in Supplemental Methods, was applied to the base of the tongue (dose: 3–6 × 10^7^ conidia per mouse). Following infection, the mice were monitored for survival over 7 or 14 days or euthanized at earlier time points for histology, immunophenotyping, BAL collection, cell isolation from lungs, or bone marrow for downstream analyses. Detailed procedures describing histology, and immunophenotyping are provided in Supplemental Methods.

For experiments involving ibrutinib or acalabrutinib treatment, mice received 25 mg/kg Ibrutinib (ChemieTek, CTPCI327) or acalabrutinib (MedChem Express, HY-17600), or vehicle (ibrutinib vehicle: 0.4% methylcellulose; acalabrutinib vehicle: 10% DMSO, 40% PEG300, 5% Tween-80, and 45% PBS) via intraperitoneal injection starting 1 day before infection and then continued daily during the experiment; ibrutinib or acalabrutinib preparation methods are described in the Supplemental Methods. For experiments to assess the potential prosurvival benefit of GM-CSF, mice received intraperitoneal doses of 5 μg recombinant GM-CSF in 100 μL PBS solution starting 6 days before infection and occurring every second day until day 8 postinfection.

### Isolation of murine neutrophils and analyses of anti-Aspergillus effector functions

Naive mice or mice at day 2 or 4 after pulmonary *Aspergillus* infection were euthanized using CO_2_ per NIH Office of Animal Care and Use guidelines. For isolation of neutrophils from the bone marrow, lungs, or BAL fluid, we followed a previously published protocol for cell isolation (105). Neutrophils were utilized in experiments to assess *Aspergillus* hyphal damage, degranulation, and oxidative burst; detailed procedures for murine neutrophil isolation and effector function analyses are described in the Supplemental Methods.

### Statistics

To determine statistical significance, 2-sided unpaired *t* tests (with or without Welch’s correction, as appropriate), 2-sided paired *t* tests, 2-sided Mann-Whitney *U* tests, 2-sided Wilcoxon tests, repeated measures or 1-way ANOVA with Dunnett’s post hoc multiple-comparison test, Friedman test with Dunn’s multiple comparison test, Kruskal-Wallis with Dunn’s multiple comparisons test, 2-way ANOVA with Šidák’s multiple comparisons test, log-rank test, or Grubb’s outlier test were used, as appropriate, using GraphPad Prism 9 software (GraphPad Software, LLC). Quantitative data are presented as means ± SEM or depicted as box and whisker plots with values ranging from minimum to maximum, and a *P* value of less-than or equal-to 0.05 was considered significant. 

### Study approval

Healthy donors, patients with lymphoma, and patients with XLA were recruited at the NIH, and enrolled in the NIH Institutional Review Board–approved protocols; the participants provided written informed consent for participation in the study. Healthy human participants were enrolled under the studies NCT00001846, and NCT01386437, while the patients with XLA were enrolled under the studies NCT00001244 and NCT00001355. For patients with lymphoma, eligible patients with primary CNS lymphoma (PCNSL) were enrolled under the study NCT02203526, while patients with aggressive B cell lymphomas with secondary involvement of the CNS (sCNSL), untreated or relapsed/refractory disease, or untreated B cell lymphoma with CNS involvement were enrolled under the study NCT03964090. The patients with lymphoma enrolled under the acalabrutinib study NCT04002947 included participants with untreated diffuse large B cell lymphoma.

The studies involving mice were carried out according to the protocols approved by the NIAID (protocol: LCIM14E) and Memorial Sloan Kettering Cancer Center (protocol: 13-07-008) Animal Care and Use Committees. 

### Data availability

All sequencing data have been deposited to the Gene Expression Omnibus (GSE243974). Publicly available, previously published data were sourced from the Gene Expression Omnibus (GEO) database (accession number GSE145033) (49, 50) to analyze *BTK* transcript levels in human neutrophils; the detailed procedure is provided in Supplemental Methods. All the raw data for the depicted plots within the article and supplemental material are provided in the Supporting Data Values file.

## Author contributions

JVD, MAZ, ALW, MAA, AD, GW, NS, MP, MRJ, JKL, LMF, and MSL performed experiments and analyzed the data. JVD, MAZ, ALW, MAA, TLL, TMH, and MSL designed experiments. MR, GU, JREB, IF, LMS, SMH, WHW, and MSL designed clinical protocols, provided clinical care, and referred patients. JKL, RAC, LMF, ESC, WNK, DY, GC, JEB, MJK, TLL, and TMH provided key reagents and expertise. MSL conceived and supervised the project. JVD, MAZ, ALW, and MSL wrote the final manuscript.

## Supplementary Material

Supplemental data

undefined

Unedited blot and gel images

Supporting data values

## Version 1 05/02/2024

In-Press Preview

## Version 2 06/17/2024

Electronic publication
