# Supplementary material for: BTK drives neutrophil activation for sterilizing antifungal immunity
Source: J Clin Invest. 2024 May 2;134(12):e176142. doi: 10.1172/JCI176142 (PMC11178547; doi:10.1172/JCI176142)
Supplement: Supplemental data [file jci-134-176142-s066.pdf]

Supplemental Figures

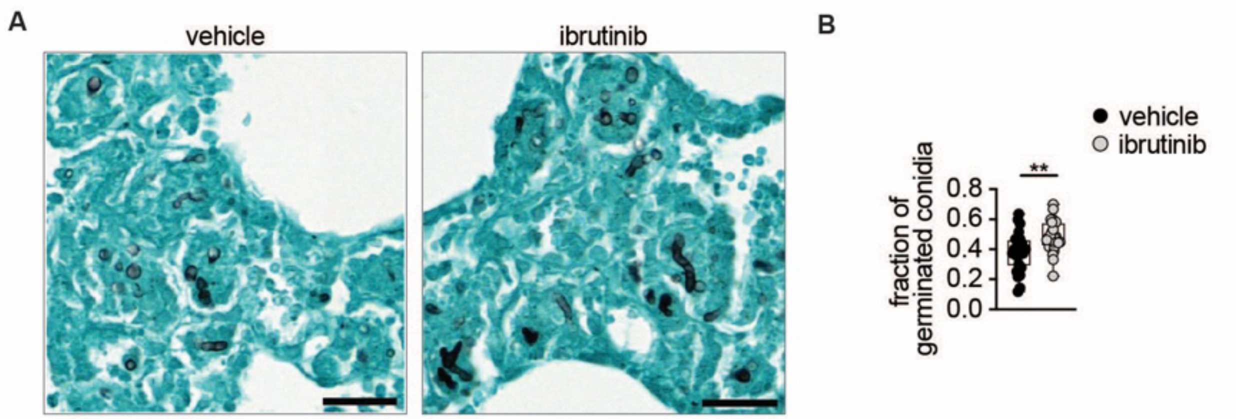

Supplemental Figure 1. Ibrutinib-treated mice exhibit a greater proportion of conidia germinating into hyphae in the lung parenchyma than control mice. (A) Grocott's methenamine silver (GMS)-stained lung sections at day 4 post-infection with 3 x 10^7 resting conidia. Scale bars: 25 μm (*n* = 4). **(B)** Quantification of the proportion of germinating *A. fumigatus* conidia. Each dot depicts an individual affected region of the lung; 5-10 such areas were randomly selected per mouse (*n* = 4 mice) and germinated conidia were enumerated. $^{**}P<0.01$, determined using two-sided unpaired *t* test (B).

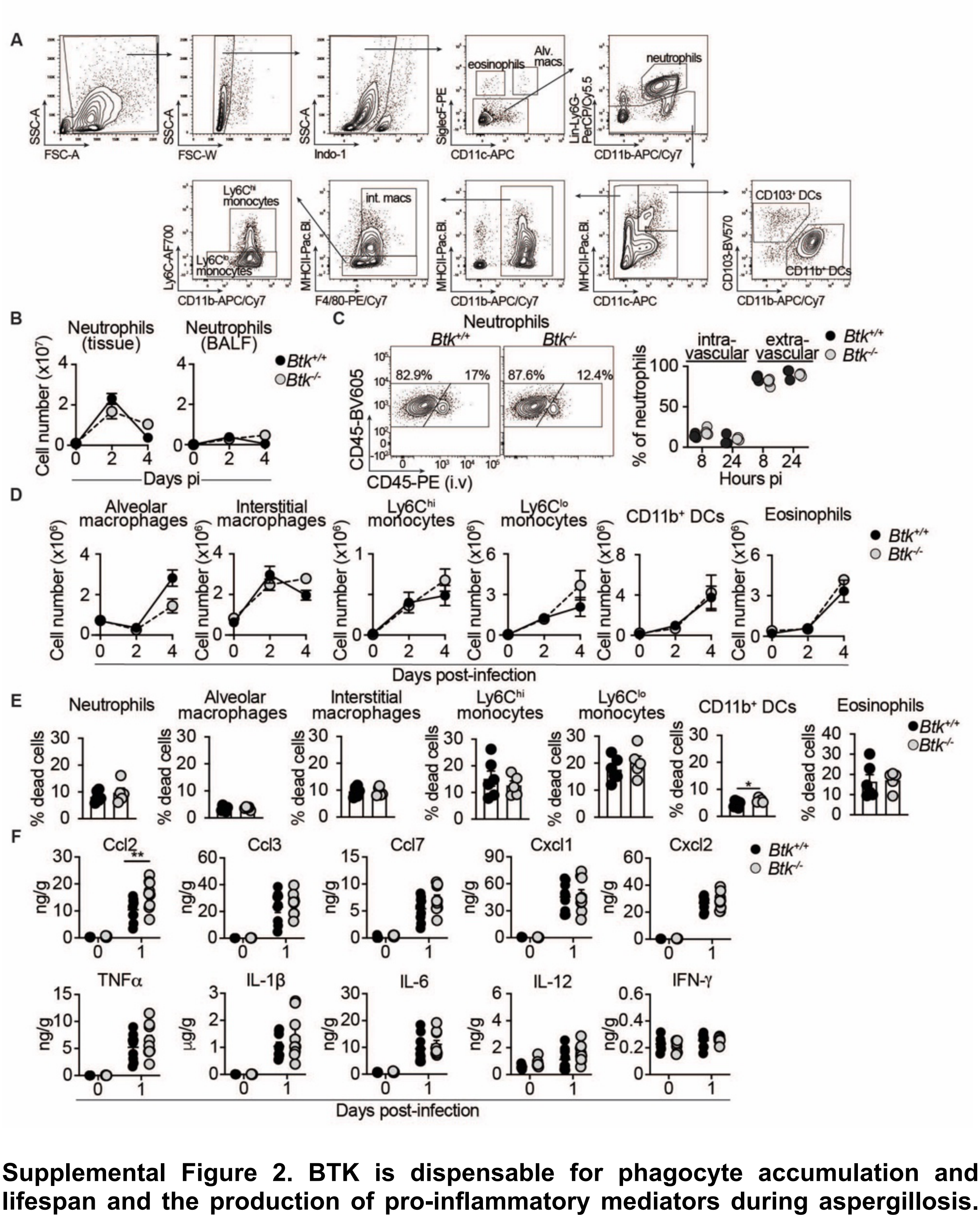

Supplemental Figure 2. BTK is dispensable for phagocyte accumulation and lifespan and the production of pro-inflammatory mediators during aspergillosis. **(A)** Gating strategy for flow cytometry-based identification of myeloid and lymphoid cells in infected lungs at day 2 post-infection. **(B-C)** Numbers of live neutrophils in lung and bronchoalveolar lavage fluid (BALF) of *Aspergillus*-infected mice at the indicated days post-infection (*n* = 3-6). **(C)** Representative FACS plots (left) and summary data (right) of

intravascular and extravasated pulmonary neutrophils of *Aspergillus*-infected mice at the indicated times post-infection (*n* = 3-4). **(D)** Numbers of live myeloid phagocyte subsets in the lung of *Aspergillus*-infected mice at the indicated days post-infection (*n* = 3-6). **(E)** Percentage of dead cells for the indicated myeloid phagocyte subsets in the lung of *Aspergillus*-infected mice at day 2 post-infection (*n* = 5-6). (**F)** Concentrations of the indicated cytokines and chemokines in the mouse lung by Luminex-based assay at steady state and day 1 post-infection (*n* = 6-9). Lin: lineage (CD3/CD19/NK1.1). Quantitative data are means ± SEM. **P*<0.05, ***P*<0.01, determined using two-sided unpaired *t* test (E) or 2-way ANOVA with Šidák's multiple comparisons test (F).

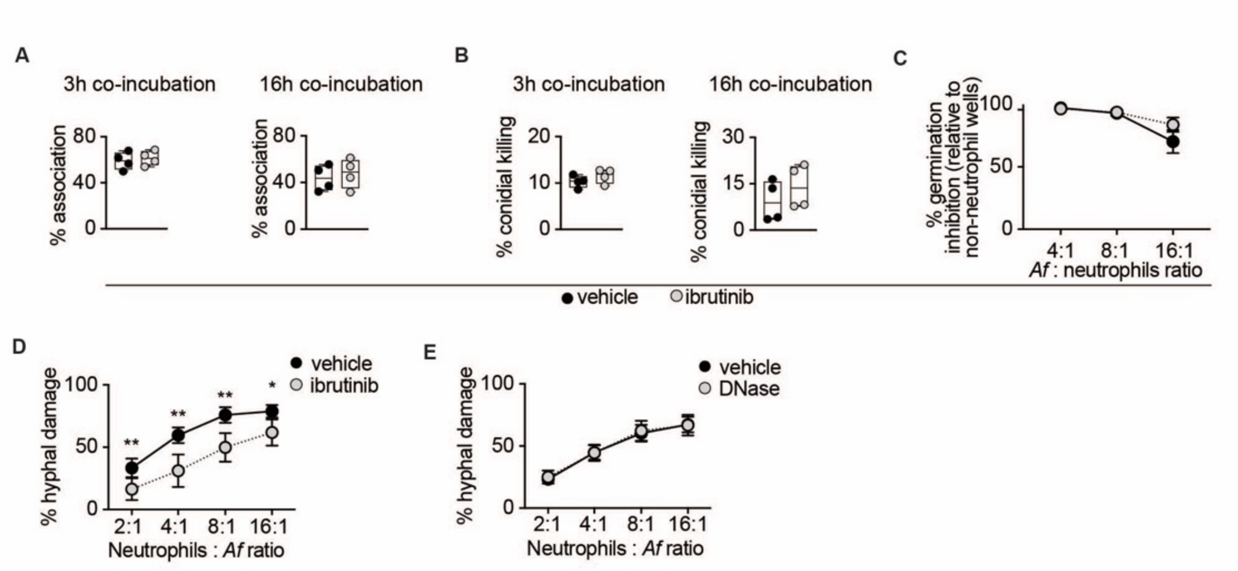

Supplemental Figure 3. Ibrutinib inhibits *Aspergillus* hyphal damage, but not conidial uptake or intracellular killing, in human neutrophils. (A-B) Healthy donor neutrophils were treated with vehicle or ibrutinib and analyzed for their ability to associate with or kill *A. fumigatus* FLARE conidia (*n* = 4). **(A)** Percentage of neutrophils associated with total (live + dead) *A. fumigatus* conidia, thus quantifying "% association". **(B)** Percentage of healthy donor neutrophils associated with dead conidia, thus quantifying "% killing". **(C)** Vehicle- or ibrutinib-treated healthy donor neutrophils were co-incubated with *A. fumigatus* conidia at the indicated effector-target ratios as indicated and the neutrophils' ability to inhibit conidial germination was quantified relative to the wells not containing neutrophils (*n* = 4). **(D)** *A. fumigatus* hyphal damage by vehicle- or ibrutinib-treated healthy donor neutrophils at the indicated effector-target ratios (*n* = 6). **(E)** Neutrophil extracellular traps are dispensable for healthy donor neutrophil-induced *A. fumigatus* hyphal damage. Neutrophils were exposed to vehicle or DNase (100U/ml) at the indicated effector-target ratios (*n* = 6). Quantitative data are mean ± SEM (C, D, E) or depicted as box and whisker plots with values ranging from minimum to maximum (A, B). Ibrutinib concentration, 250 nM. Af: *Aspergillus fumigatus*. $^*P<0.05$, $^{**}P<0.01$, determined using two-sided paired *t* test (D).

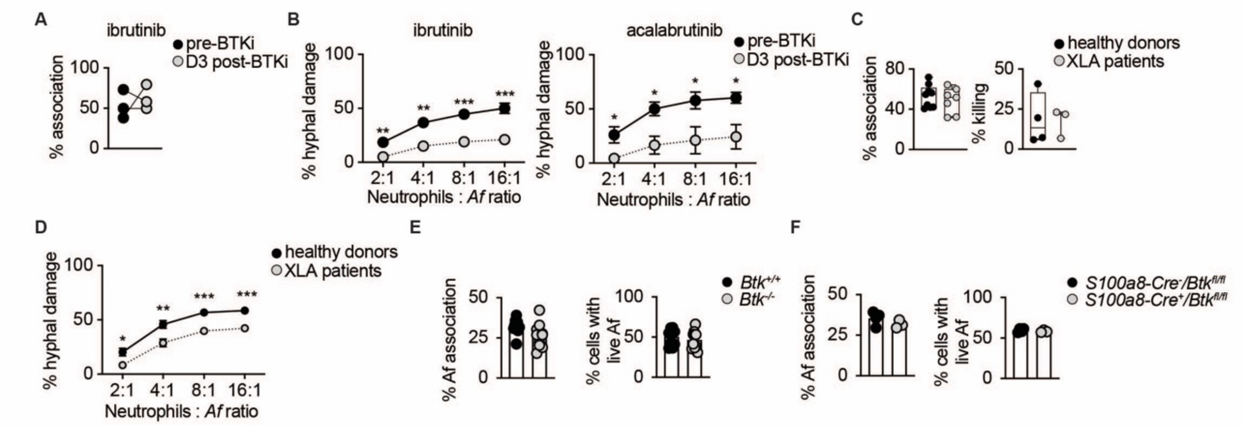

Supplemental Figure 4. BTK plays an essential role in *Aspergillus* hyphal damage but is dispensable for conidial uptake or intracellular killing by neutrophils. (A) Percentage of neutrophils isolated from lymphoma patients, before or at day 3 after treatment initiation with ibrutinib or acalabrutinib, which are associated with *A. fumigatus* FLARE reporter conidia (*n* = 3). **(B)** *Aspergillus* hyphal damage induced by neutrophils isolated from lymphoma patients, before or at day 3 after treatment initiation with ibrutinib (*n* = 8) or acalabrutinib (*n* = 5), at the indicated effector-target ratios. **(C)** Percentage of healthy donor or XLA neutrophils that contain (live + dead) *A. fumigatus* conidia, thus quantifying "% association" (left panel). Percentage of healthy donor or XLA neutrophils that contain dead conidia, thus quantifying "% killing" (right panel). (*n* = 3-9) **(D)** *A. fumigatus* hyphal damage induced by neutrophils isolated from healthy donors or from XLA patients (*n* = 9), at the indicated effector-target ratios. **(E)** WT and *Btk*$^{-/-}$ mice were infected with *A. fumigatus* (*Af*) FLARE reporter conidia and at day 2 post-infection, neutrophils were examined for their ability to associate with or kill conidia in the lung (*n* = 9-10). **(F)** Percentage of bone marrow neutrophils from the indicated strains that contain (live + dead) *A. fumigatus* conidia, thus quantifying "% association" (left panel). Percentage of bone marrow neutrophils from the indicated strains that contain dead conidia, thus quantifying "% killing" (right panel) (*n* = 3-4). Quantitative data are mean ± SEM. Box and whisker plots depict values ranging from minimum to maximum (C). BTKi: BTK inhibitor; XLA: X-linked agammaglobulinemia; Af: *Aspergillus fumigatus*. *P<0.05, **P<0.01, ***P<0.001, determined using two-sided paired *t* test (B), or two-sided unpaired *t* test (D).

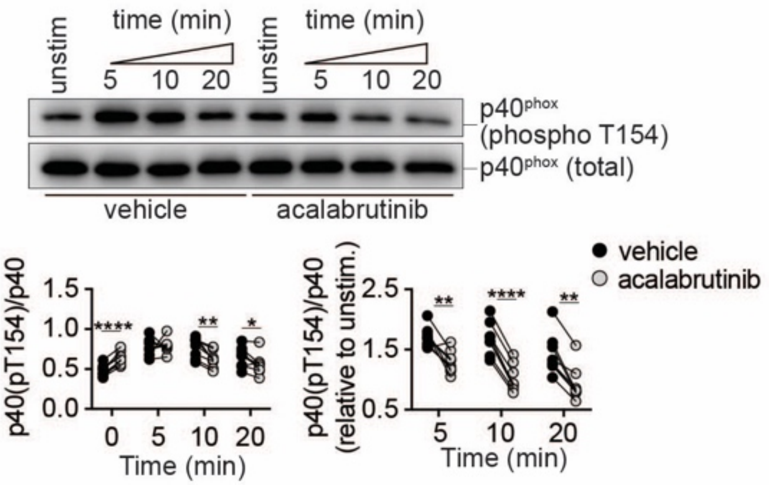

Supplemental Figure 5: Acalabrutinib inhibits p40^{phox} phosphorylation in human neutrophils. Immunoblot analysis of p40^{phox} phosphorylation (at T154) in human neutrophils upon stimulation with serum-opsonized heat-killed *Aspergillus* conidia at the indicated time points. Representative immunoblot images (top panels) and quantified pixel density values (lower panels) are shown. The ratio of pixel density for phosphorylated p40^{phox} vs. total p40^{phox} (bottom left) and fold-change of the ratios (i.e., phosphorylated p40^{phox} to total p40^{phox}) relative to unstimulated neutrophils (bottom right) are shown. Each dot depicts an individual healthy donor (n = 8). Acalabrutinib concentration, 2.5 μM. *P<0.05, **P<0.01, ****P<0.0001, determined using two-sided paired t test, or two-sided Wilcoxon test (20 min, bottom right panel).

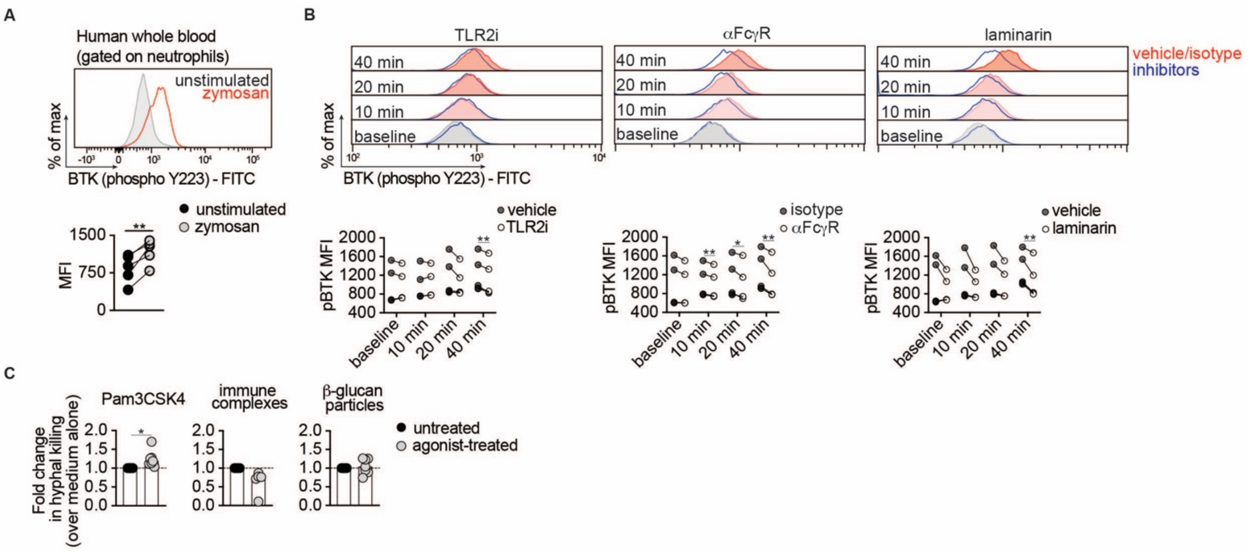

Supplemental Figure 6. Engagement of pattern recognition receptors activates BTK signaling and promotes anti-*Aspergillus* functions of healthy donor neutrophils. (A) Zymosan stimulates BTK phosphorylation in neutrophils during whole-blood stimulation. Representative FACS histogram depicts BTK phosphorylation on live neutrophils in whole human blood (top panel). Quantitative summary of phosphorylated BTK mean fluorescence intensity (MFI) (bottom panel). **(B)** TLR2, FcγR or Dectin-1 inhibition leads to abrogated BTK phosphorylation in healthy human neutrophils upon stimulation by serum-opsonized heat-killed *Aspergillus* conidia. Representative FACS histograms (upper panels) and mean fluorescence intensity (MFI) summary data (lower panels) for phosphorylated BTK (at Y223) in healthy donor neutrophils at baseline and at the indicated timepoints after stimulation with serum-opsonized heat-killed *Aspergillus* conidia, in the presence of vehicle or inhibitors, in the case of TLR2 (left panels), Dectin-1 (right panels), or isotype and blocking antibodies, in the case of FcγR (middle panels) (n = 5). **(C)** TLR2 ligation enhances damage of serum-opsonized *A. fumigatus* hyphae by healthy human neutrophils. Neutrophils were added at an effector:target ratio of 8:1 (n = 5-7). Data depict fold change in hyphal killing relative to the "medium alone" neutrophil controls, for each of the indicated agonists. Each dot represents an individual healthy donor. *P<0.05, **P<0.01 determined using two-sided paired t test (A, B) or two-sided Welch's t test (C).

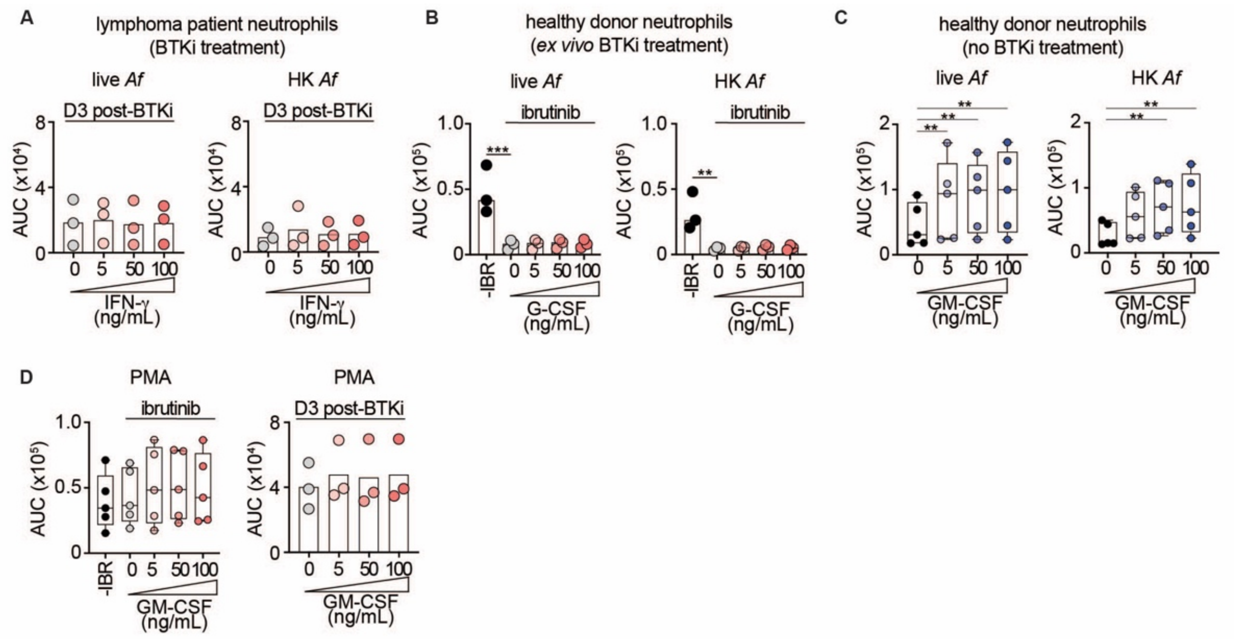

Supplemental Figure 7. GM-CSF boosts human neutrophil ROS production upon *Aspergillus* stimulation, while IFN-γ and G-CSF do not. (A) Summary data of AUC of luminol-amplified chemiluminescence RLU (expressed as AUC) in neutrophils isolated from ibrutinib- or acalabrutinib-treated lymphoma patients that were stimulated with serum-opsonized live or heat-killed (HK) *A. fumigatus* (*Af*) conidia, in the presence of increasing concentrations of IFN-γ (n = 3). **(B)** Summary data of AUC of luminol-amplified chemiluminescence RLU (expressed as AUC) in vehicle- or ibrutinib-treated healthy donor neutrophils that were stimulated with serum-opsonized live or HK *Af* conidia, in the presence of increasing G-CSF concentrations (n = 3). **(C)** Summary data of AUC of luminol-amplified chemiluminescence RLU (expressed as AUC) in healthy donor neutrophils that were stimulated with serum-opsonized live or HK *Af* conidia, in the presence of increasing GM-CSF concentrations (n = 5). The baseline GM-CSF-unexposed AUC data are also depicted for the baseline "-IBR" data in Figure 7B, as the experiments with/without ibrutinib and GM-CSF were conducted at the same time. **(D)** Summary RLU AUC data for vehicle- or ibrutinib-treated healthy donor neutrophils (left; n = 5) or in neutrophils isolated from ibrutinib- or acalabrutinib-treated lymphoma patients (right; n = 3) that were stimulated with PMA in the presence of the indicated increasing GM-CSF concentrations. PMA, phorbol-12-myristate-13-acetate; IFN-γ: interferon γ; G-CSF: granulocyte colony-stimulating factor. Ibrutinib concentration, 250 nM. Each dot represents an individual healthy donor or patient. BTKi: BTK inhibitor. **P<0.01, ***P<0.001 determined using repeated measures one-way ANOVA with Dunnett's multiple comparisons test (B; C, live *Af)* or Friedman's test with Dunn's multiple comparison test (C, HK *Af*).

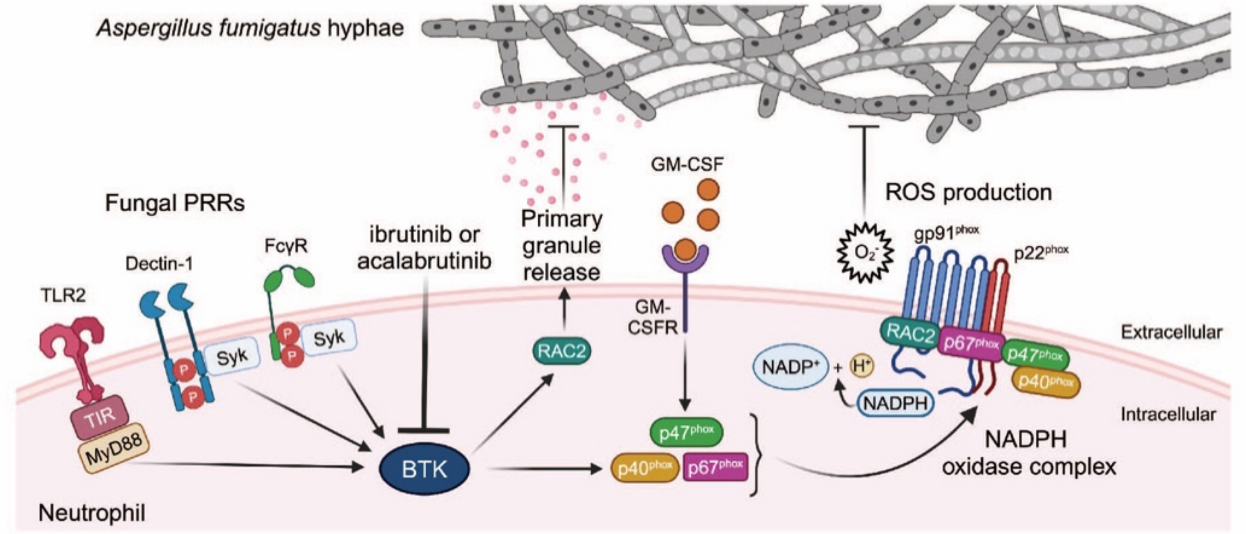

Supplemental Figure 8. Model of BTK-dependent promotes neutrophil activation in response to *Aspergillus fumigatus*. Recognition of *Aspergillus fumigatus* PAMPs by the fungal PRRs TLR2, Dectin-1 and/or FcγR leads to phosphorylation of BTK. Once activated, BTK mediates ROS production through phosphorylation of the $p40^{phox}$ subunit of the NADPH oxidase complex, triggering translocation of the cytosolic components ($p40^{phox}$, $p47^{phox,}$ and $p67^{phox}$) to complex with $gp91^{phox}$, $p22^{phox}$ and RAC2 at the phagosomal membrane. The activated NADPH oxidase complex catalyzes the oxidation of NADPH, leading to ROS generation. BTK also promotes the activation of RAC2 to drive the release of primary granules harboring anti-microbial molecules such as myeloperoxidase. Both neutrophil effector functions, i.e. ROS production and primary granule release are suppressed by the BTK inhibitors ibrutinib and acalabrutinib. The suppressed ROS production can be rescued with the FDA-approved cytokine granulocyte macrophage-colony stimulating factor (GM-CSF), which acts through phosphorylation of $p47^{phox}$ to bypass the BTK inhibition-induced neutrophil deficit in ROS production. PAMPs: pathogen-associated molecular patterns; BTK: Bruton's tyrosine kinase; PRRs: pattern recognition receptors; TLR2: toll-like receptor 2; FcγR: Fc receptors for IgG; ROS: reactive oxygen species; NADPH: nicotinamide adenine dinucleotide phosphate oxidase; RAC2: Rac Family Small GTPase 2. The illustration was created with BioRender.com.

Supplemental Table 1. Demographic, clinical, and research evaluation of the 49 lymphoma patients included in the present study. Refer to the submitted Excel file.

Supplemental Table 2. Demographic, genetic, and research evaluation of the 9 X-agammaglobulinemia patients included in the present study. Refer to the submitted Excel file.

Supplemental Methods

Aspergillus fumigatus conidia growth and harvesting

Aspergillus fumigatus (*Aspergillus*) B-5233 strain was a gift from Dr. Kwon-Chung at NIAID, NIH. The Af293 and Af293::dsRed *Aspergillus* strains have been described previously (1, 2). The B-5233 clinical isolate was used for most functional studies, unless specified otherwise. After 5-9 days of culture on malt extract agar slants at 37°C, the conidia were harvested using 0.01% Tween-20 (G-Biosciences, Cat# 786-518) with PBS (Corning, Cat# 21-040-CM), filtered through a 40 μm nylon cell strainer, washed twice with 0.01% Tween-20 in PBS and counted.

Generation of the *A. fumigatus* B-5233:mRFP strain

To generate the *A. fumigatus* B-5233:mRFP strain, protoplasts were generated using *Trichoderma harzianum* lysing enzyme (MilliporeSigma). A construct consisting of the *gpdA* promotor from *Aspergillus nidulans,* mRFP, and the *ptrA* resistance marker (3) was inserted into the genome ectopically via polyethylene glycol mediated transformation (4, 5). Protoplasts were selected for on media containing 4 μg/ml pyrithiamine hydrobromide (MilliporeSigma). Pyrithiamine resistant conidia were screened by flow cytometry, comparing red fluorescence in the ECD-A channel to that of B-5233 wildtype, and the W72310:mRFP control strain (6). Growth of the *A. fumigatus* B-5233:mRFP strain was comparable to the wild-type *A. fumigatus* B-5233 strain by quantifying their radial growth on gut microbiota medium (GMM) agar.

Generation of fluorescent *Aspergillus* reporter (FLARE) conidia

Af293::dsRed, or B-5233::mRFP conidia were coupled with streptavidin-Alexa Fluor 633 as described previously (2). Briefly, the conidia were resuspended at $5x10^8$ conidia/ml in 50 mM sodium carbonate, pH 9.5 and were incubated with 0.5 mg/ml biotin-XX-SSE (6-((6-((biotinoyl)amino)hexanoyl)-amino) hexanoic acid), sulfosuccinimidyl ester, sodium salt (Thermo Fisher Scientific) for 2 hours at room temperature with continuous shaking. Subsequently, the biotin-coupled conidia were washed twice with 0.1M Tris-HCl, pH 8, resuspended in PBS containing 0.02 mg/ml streptavidin-Alexa Fluor 633 (Thermo Fisher Scientific) and incubated for 30 minutes at room temperature with continuous shaking, then were washed once with PBS containing 0.01% Tween-20, and used in downstream applications.

Ibrutinib and acalabrutinib drug preparation

Ibrutinib (MedChemExpress, Cat# HY-10997) and acalabrutinib (MedChemExpress, Cat# HY-17600) were prepared at a 50 mM stock in dimethyl sulfoxide (DMSO) (Milli-poreSigma, Cat# D2650). Stocks were further diluted in appropriate reagents for the re-spective functional assay at the specified concentrations. For the treatment of mice, ibru-tinib suspension was prepared in 0.4% methyl cellulose (MilliporeSigma, Cat# M0262-250G) and acalabrutinib suspension was prepared using stepwise addition of 10% DMSO (MilliporeSigma, Cat# D2650), 40% PEG300 (MedChemExpress, Cat# HY-Y0873), 5% Tween-80 (MedChemExpress, Cat# HY-Y1891) and 45% PBS (Corning, Cat# 21-040-CM) to deliver a dose of 25 mg/kg/day, as specified below under the "Mouse pulmonary *Aspergillus* infection" section.

Association and intracellular killing of *Aspergillus fumigatus* FLARE conidia by human neutrophils

Human neutrophils from healthy volunteers or patients were isolated from whole blood, as above, and were adjusted to 2 x 10^6/ml in RPMI-1640 (Corning, Cat# 10-041-CV) with 1% penicillin/streptomycin (P/S) (Corning, Cat# 30-002-CI). FLARE Af293-dsRed, and FLARE B-5233-mRFP conidia were harvested and labeled as described above and were opsonized with 20% human serum. Neutrophils were pretreated with ibrutinib or vehicle control and then co-incubated with the opsonized conidia for 3 hours or 16 hours at a 1:1 multiplicity of infection (MOI). Subsequently, the neutrophils were stained using a human anti-CD15 antibody (clone HI98, BioLegend, Cat# 301908) and analyzed by flow cytometry to define $CD15^+$ neutrophils that are associated with conidia (dsRed/$mRFP^-$ $AF633^+$ or dsRed/$mRFP^+$ $AF633^+$), neutrophils that contain live ($dsRed^+$ $AF633^+$ or $mRFP^+$ $AF633^+$) or killed conidia ($dsRed^-$ $AF633^+$ or $mRFP^-$ $AF633^+$), and bystander neutrophils that have not engaged with conidia ($dsRed^-$ or $mRFP^-$ and $AF633^-$).

***Aspergillus fumigatus* conidial germination inhibition by human neutrophils**

The *ex vivo* efficacy of neutrophil inhibition of conidial germination was assessed using a 2,3-bis (2-methoxy-4-nitro-5-sulfophenyl)-5 [(phenylamino) carbonyl]-sH-tetrazolium hydroxide (XTT) based colorimetric assay. Briefly, human neutrophils isolated from whole blood were resuspended at 8x10^6/ml in RPMI medium containing 10% heat-inactivated fetal bovine serum (R&D Systems, Cat# S11550H), and penicillin/streptomycin. Neutrophils were pretreated with ibrutinib or vehicle control for 30 minutes, and, subsequently, were added to 96-well cell culture plates containing opsonized *Aspergillus* conidia at

different effector to target ratios (neutrophil to conidia ratios 4:1, 8:1, 16:1). The neutrophil-*Aspergillus* co-cultures were incubated at 37°C for 17 hours, in a humidified incubator containing 5% CO_2. After 17 hours, the neutrophils were hypotonically lysed, and the conidial germination was assessed by treating the *Aspergillus* with 0.5 mg/ml XTT (Fisher Scientific, Cat# J61726) and 40 µg/ml 2,3-dimethoxy-5-methyl-1,4-benzoquinone coenzyme Q (Alfa Aesar, Cat# B24777) for 1 hour at 37°C. Colorimetric conversion of XTT into formazan by *Aspergillus* was recorded by measuring absorbance at 450 nm with background correction at 660 nm using a microplate reader (Agilent, BioTek Synergy H1). Percentage germination inhibition of conidia in wells containing neutrophils was enumerated relative to the wells containing *Aspergillus* without neutrophils.

***Aspergillus fumigatus* hyphal damage by neutrophils**

The killing efficacy of neutrophils against hyphae was assessed by the XTT-based colorimetric assay. The day before the experiment, *Aspergillus* conidia were harvested, counted, and adjusted to concentration of $2x10^6$/ml in RPMI-1640 (Corning, Cat# 10-041-CV) & 1% penicillin-streptomycin (Corning, Cat# 30-002-CI) with 20% fetal bovine serum (R&D Systems, Cat# S11150). Conidial suspensions were added at $2x10^5$, $1x10^5$, $0.5x10^5$ or $0.25x10^5$ conidia per well of a flat-bottom 96-well plate and placed in a 37°C incubator with 5% CO_2 for 12 hours to allow for hyphal formation. After the 12-hour incubation, human neutrophils from healthy volunteers or patients were isolated from whole blood, counted and adjusted to $8x10^6$ cells/ml in RPMI-1640 + 1% P/S. The hyphae were opsonized with 20% human serum from healthy donors for 30 minutes and co-incubated with $4x10^5$ neutrophils per well (to create effector to target ratios of 2:1, 4:1, 8:1, and 16:1) for

2 hours. 6 technical replicates were used for each target ratio. Non-neutrophil-containing hyphal wells in triplicate, were used as reference controls. After spinning the plates at 3200*g* for 10 minutes, the supernatants were stored for granule analysis by ELISA, and the remaining neutrophils were hypo-tonically lysed with deionized water. The remaining hyphae were washed with deionized water and incubated with 0.5 mg/ml XTT (Fischer Scientific, Cat# J61726) and 40 µg/ml 2,3-dimethoxy-5-methyl-1,4-benzoquinone coenzyme Q0 (Alfa Aesar, Cat# B24777) for 1 hour at 37°C. Absorbance was read at 450 nm wavelength with 660 nm wavelength correction using a microplate reader (Agilent, BioTek Synergy H1), and percentage hyphal damage was calculated relative to non-neutrophil-containing wells.

To assess the role of neutrophil extracellular traps in *A. fumigatus* hyphal damage, opsonized hyphae were co-incubated with vehicle or 200 U/ml DNase (Roche, Cat# 4716728001), and $4x10^5$ healthy donor neutrophils per well were added. Hyphal damage was assessed as described above.

To assess the role of TLR2 or Dectin-1 agonists in boosting *A. fumigatus* hyphal damage by neutrophils, the neutrophils were resuspended at $8x10^6$ cells/ml in RPMI-1640 + 1% P/S, and were treated with 10 μg/mL Pam3CSK4 (TLR2 agonist; Invivogen, Cat# tlrl-pms) or 0.2 mg/mL β-glucan particles (Dectin-1 agonist; (gift from Dr. Yamanaka at Tokyo University of Pharmacy and Life Sciences, Tokyo, Japan) for 30 minutes at 37°C. For FcγR stimulation, $4x10^5$ neutrophils were added per well to wells containing immobilized immune complex for 30 minutes at 37°C. To immobilize the immune complexes, wells of flat-bottomed 96-well were coated with 20 µg/ml human serum albumin (MilliporeSigma, Cat# A1653) in 50 mM carbonate buffer, pH 9.4 (MilliporeSigma, Cat# S7795)

overnight at 4°C, washed with PBS + 0.05% Tween-20, blocked with 10% BSA (Fisher Scientific, Cat# BP9705-100) in PBS for 1 hour at room temperature and coated with 10 µg/ml anti-HSA antibody (Invitrogen, Cat# MA5-29022) for one additional hour. Subsequent to the agonist treatment, the primed neutrophils were added atop opsonized hyphae at $4x10^5$ neutrophils per well, and the hyphal damage was assessed as described above.

Measurement of human neutrophil degranulation in response to *Aspergillus fumigatus* hyphae

The supernatants of human neutrophils co-incubated with preformed hyphae, were harvested and used for downstream enzyme-linked immunosorbent-assay (ELISA) experiments to measure neutrophil degranulation response against *Aspergillus* hyphae. We used the human myeloperoxidase DuoSet ELISA kit (R&D Systems, Cat# DY3174), human lactoferrin ELISA kit (Abcam, Cat# ab108882) and human MMP-9 DuoSet ELISA kit (R&D Systems, Cat# DY911) according to the manufacturer's instructions to measure myeloperoxidase (primary granules), lactoferrin (secondary granules) and MMP-9 (tertiary granules) respectively. Absorbance of the ELISA plates was read at 450 nm wavelength with 570 nm wavelength correction using a microplate reader (Agilent, BioTek Synergy H1).

Flow cytometric evaluation of neutrophil reactive oxygen species (ROS) production using dihydrorhodamine 123

Human neutrophils from healthy volunteers or patients were isolated from whole blood, and adjusted to 6.25×10^5 per ml in DHR buffer (0.1% of bovine serum albumin (Fisher Scientific, Cat# BP9705-100) and 0.2% of 0.5M EDTA pH 8.0 (Corning, Cat# 46-034-CI) in 1x HBSS without Ca, Mg, or phenol red (Corning, Cat# 21-022-CV, sterile filtered). Neutrophils were then pretreated with ibrutinib or acalabrutinib (250 nM) or vehicle control for 30 minutes at 37°C. 2.5×10^5 neutrophils were used for each condition and were stimulated with opsonized live *Aspergillus fumigatus (Af)* conidia (1×10^8/ml), opsonized heat-killed *Af* conidia (heat killed at 99°C for 30 minutes) (1×10^8/ml), opsonized zymosan (1 mg/ml) (MilliporeSigma, Cat# Z4250), phorbol 12-myristate 13-acetate (PMA) (MilliporeSigma, Cat# P8139) or control in the presence of an anti-human CD15 antibody (clone HI98, BioLegend, Cat# 301908) and 125 µM dihydrorhodamine 123 (Invitrogen, Cat# D632). After 30 minutes of stimulation, stimulation was halted via placing the samples on ice, and the fluorescence was immediately measured in the FITC channel via flow cytometry. $CD15^+$ neutrophils were analyzed using a BD LSR Fortessa II instrument using BD FACS Diva acquisition software and analyzed using FlowJo software (BD Biosciences, Franklin Lakes, NJ).

Measurement of neutrophil ROS production using luminol

Neutrophils were isolated and resuspended at 1.1×10^6/ml in luminol assay medium (Seahorse XF RPMI media without Phenol Red (Agilent, Cat# 103576-100), 2 mM L-glutamine (Agilent, Cat# 103579-100), 10 mM HEPES (Quality Biological, Cat# 118-089-721), and 11 mM glucose (Agilent, Cat# 103577-100)). For ex vivo treatment experiments, ibrutinib (0.25 µM or 2.5 µM), ibrutinib with 5, 50 or 100 ng/ml granulocyte macrophage-colony

stimulating factor (GM-CSF; PeproTech, Cat# 300-03) or vehicle were added to the cell suspension and incubated at 37°C for 15 minutes, after which 55 μM luminol reagent (MilliporeSigma, Cat# A8511) was added and incubated at 37°C for a further 15 minutes to activate the reagent. Stimuli were added in a 96-well high binding half area white plate (Greiner Bio-One, Cat# 675074), including opsonized live and heat-killed *Aspergillus* (heat-killed at 99°C for 30 minutes) at a conidia:neutrophil MOI of 40:1, 1 μg/ml Pam3CSK4 (Invivogen, Cat# TLRL-PMS), coating of plate with 20 μg/ml human serum albumin for immune complexes (MilliporeSigma, Cat# A1653), and 0.2 μg/ml β-glucan (gift from Dr. Yamanaka at Tokyo University of Pharmacy and Life Sciences, Tokyo, Japan) after which cell suspension was added and chemiluminescence was read over 2 hours using a microplate reader (Agilent, BioTek Synergy H1).

Analysis of *BTK* expression in human neutrophils

To assess *BTK* transcript levels in human neutrophils, previously published data were sourced from the Gene Expression Omnibus (GEO) database (accession number GSE145033) (7); these data were acquired using RNA from healthy human neutrophils isolated from 10 male and 10 female donors (7). The raw read counts were downloaded from the GEO database and relevant attributes, including "Gene Symbol" and "Transcript Length" were retrieved using biomaRt (8, 9). Subsequently, expression was determined using edgeR (10).

Intracellular staining and assessment of BTK phosphorylation in neutrophils

Neutrophils from whole blood were harvested via negative immunomagnetic separation using the EasySep™ Direct Human Neutrophil Isolation Kit (STEMCELL Technologies, Cat# 19666) per kit instructions and resuspended at 5.55×10^6/ml in luminol assay medium (Seahorse XF RPMI media without Phenol Red (Agilent, Cat# 103576-100), 2mM L-glutamine (Agilent, Cat# 103579-100), 10mM HEPES (Quality Biological, Cat# 118-089-721), and 11mM glucose (Agilent, Cat# 103577-100) containing LIVE/DEAD Fixable Blue Dead Cell Stain (Thermo Fisher Scientific, Cat# L34962). 5×10^5 cells were stimulated with one of the following: 0.4 µg/ml phorbol 12-myristate 13-acetate (PMA) (MilliporeSigma, Cat# P8139), 1 mg/ml opsonized zymosan (MilliporeSigma, Cat# Z4250), 0.2 µg/ml β-glucan (gift from Dr. Yamanaka at Tokyo University of Pharmacy and Life Sciences, Tokyo, Japan), 2×10^8/ml opsonized live *Aspergillus* conidia, or 2×10^8/ml opsonized heat-killed *Aspergillus* conidia (heat killed at 99°C for 30 minutes).

To assess BTK phosphorylation in response to immune complexes, 5×10^5 neutrophils were added to flat bottom wells prepared in the following manner: wells were coated with 20 µg/ml human serum albumin (MilliporeSigma, Cat# A1653) in 50mM carbonate buffer, pH 9.4 (MilliporeSigma, Cat# S7795) overnight at 4°C, washed with PBS + 0.05% Tween-20, blocked with 10% BSA (Fisher Scientific, Cat# BP9705-100) in PBS for 1 hour at room temperature and coated with 10 µg/ml anti-HSA antibody (Invitrogen, Cat# MA5-29022) for one additional hour.

Cells were stimulated for the specified duration, and after the completion of final stimulation, cells were fixed with warm 1.6% paraformaldehyde (PFA) (Thermo Fisher Scientific, Cat# J19943-K2) for 10 minutes at 37°C and permeabilized with 100% ice cold methanol (MilliporeSigma, Cat# 179337). Cells were then washed with PBS and FACS

buffer, incubated with an anti-BTK phospho-Y223 antibody (clone EP420Y, Abcam, Cat# ab68217), anti-BTK antibody (clone 53/BTK, BD Biosciences, Cat# 611117), and mouse IgG (Thermo Fisher Scientific, Cat# 10400C), overnight at 4°C in the dark. Cells were then incubated with an anti-CD16 antibody (clone 3G8, BD Biosciences, Cat# 555404) and mouse IgG for 40 minutes on ice, followed by 3 washes with FACS buffer, and fixing with cold 3.2% PFA. Cells were acquired on a BD LSR Fortessa II instrument using BD FACS Diva acquisition software and analyzed using FlowJo software (BD Biosciences, Franklin Lakes, NJ). To assess the impact of TLR2 or Dectin-1 inhibition on BTK phosphorylation upon stimulation with serum-opsonized heat-killed *Aspergillus* conidia, 5×10^5 neutrophils were treated with 200 μM TLR2-IN-C29 (SelleckChem, Cat# S6597) or 50 μg/mL laminarin (Invivogen, Cat# tlrl-lam) or vehicle for 30 minutes at 37°C. To assess the impact of FcγR inhibition, $5x10^5$ neutrophils were treated with a combination of 10 μg/mL anti-human CD16 monoclonal antibody (Thermo Fisher Scientific, Cat# 16-0167-82), and anti-human CD64 antibody (Biolegend, Cat# 305002) or IgG1 isotype control (Thermo Fisher, Cat# 16-4714-82) for 30 minutes at 37°C. After 30 minutes, the cells were stimulated with $2x10^6$ opsonized heat-killed *Aspergillus* conidia for different time points and were then analyzed as above.

For zymosan stimulation in human whole blood, 1 mg/ml zymosan was directly added to heparinized whole blood, along with LIVE/DEAD fixable blue stain (Thermo Fisher Scientific, Cat# L34962) and a mouse antibody against human CD15 (clone HI98, BioLegend, Cat# 301908) which were added, mixed, and incubated at 37°C for 20 minutes. Subsequently, the cells in whole blood were fixed by adding 2 ml of pre-warmed Phosflow Lyse/Fix buffer (BD Biosciences, Cat# 558049) for 10 minutes in a

water bath at 37°C with intermittent mixing. After centrifugation at 350*g* for 6 minutes, Lyse/Fix buffer was removed, and the cells were washed with ice-cold phosphate-buffered saline (PBS). Cells were then permeabilized, stained, and analyzed as above.

Evaluation of NADPH oxidase subunits' phosphorylation in neutrophils

Neutrophils were stimulated, and NADPH oxidase subunit activation was analyzed as previously described by El-Benna et al. (11). Briefly, $1x10^6$ neutrophils in 1x HBSS (Corning, Cat# 21-022-CV) were pretreated with 2.5 µM ibrutinib, 2.5 µM acalabrutinib, or vehicle control, and for certain experiments with 2.5 µM ibrutinib plus 50 ng/ml GM-CSF (PeproTech, Cat# 300-03) for 30 minutes at 37°C and then stimulated with $1x10^7$ opsonized heat-killed *Aspergillus* conidia (heat killed at 99°C for 30 minutes) for indicated time points at 37°C with shaking at 120 rpm. The reaction was stopped by adding boiling 4x modified Laemmli buffer made with Laemmli sample buffer (Bio-Rad, Cat# 1610747), 10% 2-mercaptoethanol (MilliporeSigma, Cat# M6250), 1x protease inhibitor (Thermo Fisher Scientific, Cat# 78420) and 1x phosphatase inhibitor (Thermo Fisher Scientific, Cat# 78430). Samples were then lysed by boiling for 2 minutes at 99°C and stored at -80°C until use. Neutrophil lysates were then thawed, sonicated, spun down to get rid of debris and subjected to 10% SDS-PAGE. The separated proteins were then transferred to nitrocellulose (Bio-Rad, Cat# 1704158EDU) or PVDF (Bio-Rad, Cat# 1704156EDU) using Bio-Rad Trans-Blot Turbo Transfer system (Bio-Rad, Cat# 1704150EDU), which was blocked with 5% milk in Tris-buffered saline (Corning, Cat# 46-012-CM) containing 0.1% Tween-20 (G-Biosciences, Cat# 786-518) (TBS-T) for 1 hour. After blocking, the membranes were probed with one of the following primary antibodies overnight at 4°C:

anti-phospho-Tyr154-p40phox (1:2000) (Cell Signaling Technology, Inc., Cat# 4311), anti-p40phox (1:25000) (Abcam, Cat# ab76158), anti-phospho-Ser345-p47phox (1:2000) (custom antibody generously provided as a gift by Dr. El-Benna (12)), anti-p47phox(1:5000) (Abcam, Cat. #ab179457), followed by incubation with HRP-labeled goat anti-rabbit antibody (1:1000) (Cell Signaling Technology, Inc., Cat# 7074) for 1 hour at room temperature. The protein bands were revealed using Bio-Rad Clarity Western ECL Substrate (Bio-Rad, Cat# 1705061) or Azure Biosystems Radiance Plus (Azure Biosystems, Cat# AC2104) on a ChemiDoc imaging system (Bio-Rad, Cat# 12003153). Quantification was obtained by densitometry image analysis using FIJI (13).

PAK1-PDB-GST fusion protein isolation and RAC2 activation (PBD-binding) assays

The $p21^{RAC}$-binding domain (residues 70-117) of human p21-activated kinase (PAK1) constructed as a GST fusion protein in pGEX (Addgene, Cat# 12217) was expressed in BL21 (DE3, Thermo Fisher Scientific, Cat# C600003) *E. coli*. bacteria (100 ml) overnight at 37°C. After reaching Log-phase (I_{600}>0.6) bacteria were induced with 1 mM isopropyl ß-D-1-thiogalactopyranoside (IPTG) for 3 hours at 30°C then collected by centrifugation for 10 minutes at 2300*g* at 4°C, washed with cold PBS and resuspended in 1 ml lysis buffer (50 mM Tris-HCl, pH 7.5, 150 mM NaCl, 5 mM $MgCl_2$, 1 mM EDTA, 1 mM dithiothreitol (DTT), 1 mM phenylmethylsulphonyl fluoride (PMSF), 1% Protease Inhibitor Cocktail (PIC), 1mg/ml lysozyme, 20 mg/ml DNase). After incubation on ice for 10 minutes, bacteria lysates were sonicated 3 times for 30 seconds and cleared by centrifugation for 15 minutes at 9300*g* at 4°C. Supernatants were incubated with 200 µl Glutathione-

Sepharose beads for 2 hours at 4°C while tumbling. Bead-bound PAK1-PBD-GST proteins were collected by centrifugation for 2 minutes at 400*g* at 4°C, and washed 5 times with wash buffer (50 mM Tris-HCl, pH 8.0, 150 mM NaCl, 5 mM $MgCl_2$, 1 mM DTT, 1 mM PMSF, 1% PIC) (14). Beads were kept in 200 µl wash buffer supplemented with protease inhibitor cocktail at 4°C until use or frozen with 10% v/v glycerol at -80°C.

Active RAC2 pull-down assay

Human neutrophils from healthy volunteers were isolated by negative immunomagnetic separation using the EasySep™ Direct Human Neutrophil Isolation Kit (STEMCELL Technologies, Cat# 19666) per kit instructions. Cells were resuspended at $44x10^6$/ml in 1x HBSS and either 10 million cells aliquoted with HBSS media control, vehicle (DMSO) or 2.5 µM ibrutinib to pretreat the cells for 30 minutes at 37°C. Unstimulated cells were then lysed on ice using 2x assay/lysis buffer kit component (Abcam, Cat# 21162) with added protease inhibitor (MilliporeSIGMA, Cat# P8340) and phosphatase inhibitors (MilliporeSIGMA, Cat# P2850 & Cat# P0044). For cells designated to undergo stimulation, opsonized zymosan at 1 mg/ml (MilliporeSigma, Cat# Z4250) was added and stimulated for 30 minutes at 37°C with shaking at 120 rpm. After 30 minutes the cells were lysed on ice using the same 2x assay/lysis buffer kit component containing protease and phosphatase inhibitors. All cell lysates were then spun at 9300*g* at 4°C for 10 minutes after which supernatant was transferred to new tubes. 50 µl of cleared neutrophil lysates were saved with 10 ml of 6x SDS sample buffer from each sample for total RAC2 detection. Positive and negative control lysates were supplemented with 10 mM EDTA, then 10 ml of 10 mM GTPγS or 10 ml of 100 mM GDP and incubated for 8 minutes at 30°C. Nucleotide

exchange was stopped by adding 5 mM of $MgCl_2$. Purified PAK1-PBD-GST fusion protein (5-10 µg) linked to glutathione-agarose beads was added to 450 ml of neutrophil lysates and incubated for 1 hour with rotation at 4°C. Beads were centrifuged for 2 minutes at 2000*g* and washed 3 times with ice cold PBD binding buffer for RAC (25 mM Tris-HCl, pH=7.5, 40 mM NaCl, 30 mM $MgCl_2$, 1% NP-40, 1 mM DTT, 1 mM PMSF, 5 mg/ml aprotinin, 1 mg/ml leupeptin, 2mM sodium orthovanadate). PAK1-PBD-bound proteins were eluted by suspension in 50 µl 2x SDS Laemmli buffer and saved until analysis at -80°C.

Detection of PAK1-PBD-GST bound RAC2 by Western blot

25µl of protein samples were denatured by heating at 90°C for 5 minutes, followed by separation on 4-12% or 12% Bis-Tris polyacrylamide gels. Proteins were blotted onto nitrocellulose membranes using the iBlot™ 2 dry transfer system (Thermo Fisher Scientific, Cat# IB21001). Membranes were blocked by 5% milk in 0.1% Tween-20 1x TBS (TBST) for 1 hour. Primary antibodies were diluted in 5% BSA TBST (anti-RAC2, 1000x, MilliporeSIGMA, Cat# 07-604-I; anti-GAPDH, 5000x, Trevigen, Cat# 2275-PC-100) solution and incubated for overnight at 4°C. Membranes were washed in TBST and probed with HRP-conjugated secondary antibody for 30 minutes in 5000x dilution, washed again and developed with SuperSignal West Dura ECL reagent (Thermo Fisher Scientific, Cat# 34075). Images were captured with iBright™ CL1000 Imaging System (Invitrogen, model A32747).

Single-cell RNA-seq

For single cell sequencing, sodium heparin or EDTA treated blood collected from three lymphoma patients, at one day prior and three days post-acalabrutinib treatment, was utilized. White blood cells were isolated with the Erythroclear Red Blood Cell Depletion Reagent Kit (STEMCELL Technologies, Cat# 01738) and neutrophils using EasySep™ Direct Human Neutrophil Isolation Kit (STEMCELL Technologies, Cat# 19666), according to manufacturer's protocol. Cells were washed with PBS with 0.02% bovine serum albumin (BSA), and utilized for single-cell RNA-seq. To obtain single-cell gel beads-in-emulsion (GEMs), we resuspended cells at a concentration of 1000 cells/μl and loading the mix on a Chromium Comptroller Instrument (10x Genomics). Single-cell cDNAs and libraries were prepared with a Chromium Single Cell 3′ Library & Gel Bead Kit v3.1 (10x Genomics, Cat# 1000121). Quality and quantity of the cDNAs were assessed on a 4200 TapeStation (Agilent Technologies) with High Sensitivity D5000 DNA Screen Tape (Agilent, Cat# 5067-5592). Libraries were diluted to the same molarity and pooled for sequencing on a NovaSeq6000 (Illumina) sequencer.

Processing and analysis of single-cell RNA-seq data

Illumina run folders were demultiplexed and converted to FASTQ format with Cell Ranger mkfastq version 4.0.0 and Illumina bcl2fastq version 2.20. Reads were further counted and analyzed with Cell Ranger count version 4.0.0 and the refdata-gex-GRCh38-2020-A reference, to generate raw and filtered matrix files. Matrix files were imported into the R package Seurat version 4.0.1 for downstream processing. From the raw matrices, cells with a gene number between 100-2500 and a mitochondrial gene proportion < 0.1 were selected for downstream analysis. The matrices were then normalized by the

LogNormalize method. The FindVariableFeatures() function was used to select the top 2,000 variable genes, with the vst selection method. Scaling was performed by the function ScaleData() regressing out the mitochondrial gene content. Principal component analysis (PCA) and clustering were then performed on the scaled data. UMAP (version 0.2.7.0) was utilized for visualization. After cell types were identified using marker genes in the dataset corresponding to each sample, they were integrated. Genes that were shared among all datasets were identified for downstream integration. Anchors were identified with the FindIntegrationAnchors() function, and these anchors were used to integrate the cells together with the function IntegrateData(). To study neutrophil cell-state trajectories, we used the analysis toolkit Monocle3, which is implemented as an R package (version 0.2.3.0). A principal graph was learned on the UMAP projection of the cells with the learn_graph() function. To generate a pseudotime axis, the cells were then ordered with the order_cells() function. Gene scores are using the AddModuleScore() function.

Isolation of neutrophils from mouse bone marrow, lungs and bronchoalveolar lavage

Naïve mice or mice at day 2 or 4 post-pulmonary *Aspergillus* infection were euthanized using CO_2 per NIH Office of Animal Care and Use guidelines. For isolation of neutrophils from the bone marrow, we followed a previously published protocol for cell isolation (15). Briefly, cellular material was flushed from the bone marrow cavity, filtered through a 100 μm nylon cell strainer, and treated with ACK lysis buffer (Quality Biological, Cat# 118-156-721) to lyse RBCs. Subsequently, using anti-Ly6G microbeads (Miltenyi Biotec, Cat#

130-120-337), neutrophils were isolated as per manufacturer's recommendation using LS columns (Miltenyi Biotec, Cat# 130-042-401) and a QuadroMACS separator (Miltenyi Biotec, Cat# 130-090-976) set on a MACS MultiStand (Miltenyi Biotec, Cat# 130-042-303). For isolation of neutrophils from lungs, whole lungs were removed and transferred to a petri dish with 0.5 ml of 3 mg/ml collagenase type IV (Worthington-Biochem, Cat# LS004189) containing 40 U/ml DNase I recombinant (Roche Diagnostics, Cat# 4716728001) in PBS with 5% fetal bovine serum (R&D Systems, Cat# S11150). Lungs were then minced using a razor blade for approximately 5 minutes per lung to achieve a slurry of <0.5 mm pieces, transferred to a 50 ml conical tube containing 7 ml collagenase solution and incubated at 37°C, in a shaking water bath, for 1 hour at 180 rpm. Following this step, the slurry was passed through an 18G needle and then filtered through a 100 µm cell strainer. After washing, cells were subjected to RBC lysis with ACK lysis buffer (Quality Biological, Cat# 118-156-721) and two further wash and filter steps were performed. For neutrophil enrichment the cell suspension was subjected to positive immunomagnetic selection using anti-Ly6G microbeads (Miltenyi Biotec, Cat# 130-120-337). For isolation of neutrophils from bronchoalveolar lavage, lungs and trachea were surgically exposed and a small incision made in the trachea to allow insertion of a catheter (BD Biosciences, Cat# 381444). 700 µl of PBS was passed into the lungs and collected; this step was repeated a further three times to flush cells from the lower respiratory tract.

Histology

For hematoxylin and eosin (H&E) and Grocott's methenamine silver (GMS) staining, mouse lungs were harvested and placed in 10% formalin for 24 to 48 hours. Formalin

was then replaced with 70% ethanol to store until paraffin embedding. Sections of the lungs were prepared and stained with H&E and/or GMS (Histoserv Inc., Germantown, MD). For histological analysis of ibrutinib-treated mice, the animals were infected with 3 x 10^7 conidia, and the lungs were harvested at day 4 post-infection. To analyze germinating conidia, multiple regions of interest per mouse were randomly chosen and germination was enumerated. Germination frequency was reported as the fraction of conidia which exhibit germination.

Measurement of cytokines, chemokines, and β-D-glucan in mouse lung homogenates

Mouse lungs were harvested from naïve mice or at day 2 post-pulmonary *Aspergillus* infection and homogenized in PBS containing 0.5% Tween 20 and protease inhibitor cocktail (Roche, Cat# COEDTAF-RO), using an Omni-Tip™ homogenizer (Omni International, Cat# TH115-PCR). Homogenates were clarified by centrifugation and subsequent passage through a 0.22 µm filter. The cytokines and chemokines were measured using a multiplexed bead-based assay system (Luminex Corporation, Austin, TX) as described previously (16), while (1,3)-beta-D-glucan was measured using a GLUCATELL kit (Associates of Cape Cod Inc., Cat# GT-003) according to kit instructions for the end-point assay. Briefly, for Luminex analysis, individual Luminex bead sets were coupled to the analyte-specific capture antibodies according to the manufacturer's protocols, and biotinylated polyclonal antibodies were used at twice the recommended concentrations for a classical ELISA. Subsequently, in a 50 µl volume, the assay was run with 1,200 beads per set of the examined analytes, and the plates were read where >50 beads per bead

set were collected on a Luminex MAGPIX platform. The median fluorescence intensity of the beads was then measured for each individual bead and was analyzed using a 5P regression algorithm with the Millipex software. The concentrations were reported per gram of lung tissue.

Flow cytometry based immunophenotyping

In experiments involving characterization of intravascular and extravasated leukocytes, the mice were anesthetized and 2.5 µg of anti-CD45 antibody (clone 30-F11, BioLegend, Cat# 103106) was administered retro-orbitally. After 3 minutes, mice were euthanized via cervical dislocation and the lungs were collected for single cell suspension preparation, as described above. A small fraction of the suspension was fixed with 2% paraformaldehyde (PFA) for absolute leukocyte count using PE-conjugated counting beads (Spherotech, Cat# ACFP-70-5), as previously described (17). The remainder of the single cell suspension was first stained with LIVE/DEAD Fixable Blue Dead Cell Stain (Thermo Fisher Scientific, Cat# L34962) for 10 minutes on ice. Then, rat anti-mouse CD16/CD32 (clone 2.4G2, BD Biosciences, Cat# 553142) was added at 1:100 dilution together with 0.5% bovine serum albumin; cells were incubated on ice for 10 minutes. Cells were then stained using the following fluorochrome-coupled antibodies against specific cell surface, at 1:100 dilution: CD45 (clone 30-F11, BioLegend, Cat# 103155), SiglecF (clone E50-2440, BD Biosciences, Cat# 562068), Ly6G (clone 1A8, BioLegend, Cat# 127616), CD11b (clone M1/70, BioLegend, Cat# 101226), CD11c (clone N418, Thermo Fisher Scientific, Cat# 17-0114-82), Ly6C (clone AL-21, BD Biosciences, Cat# 561237), CD103 (clone 2E7, BioLegend, Cat# 121404), MHCII (clone M5/114.15.2, BioLegend, Cat#

107620), F4/80 (clone BM8, BioLegend, Cat# 123114), CD3 (clone 145-2C11, BioLegend, Cat# 100328), CD19 (clone 1D3, BioLegend, Cat# 152406), and NK1.1 (clone PK136, BioLegend, Cat# 108728). Staining was performed by incubating the cells with antibodies on ice for 30 minutes. Subsequently, the cells were washed thrice with FACS buffer (PBS containing 5% bovine serum albumin and 0.1% NaN_3), passed through 35 μm cell strainer, and then analyzed using a 5-laser BD LSR Fortessa II (BD Biosciences). Data were collected using FACS Diva (BD Biosciences, Franklin Lakes, NJ) and analyzed using FlowJo (BD Biosciences, Franklin Lakes, NJ).

Aspergillus fumigatus conidial uptake and killing *in vivo*

The reporter FLARE Af293-dsRed conidia were generated as above, described under the "Generation of fluorescent *Aspergillus* reporter (FLARE) conidia" section. Mice were infected with $5x10^7$ conidia via pharyngeal aspiration and at 24 hours post-infection, lungs were harvested, and single cell suspension was prepared for flow cytometry. Flow cytometry was performed using a 5-laser BD LSR Fortessa II (BD Biosciences, Franklin Lakes, NJ). Data were collected using FACS Diva and analyzed using FlowJo (BD Biosciences, Franklin Lakes, NJ). Leukocytes acquire dsRed and AF633 fluorescence after engulfing live FLARE conidia, i.e. $dsRed^+$ $AF633^+$, while loss of dsRed fluorescence and retention of AF633 fluorescence correlates with killing of engulfed conidia, i.e. $dsRed^-$ $AF633^+$ (2).

Aspergillus fumigatus conidial uptake and killing by murine bone marrow neutrophils *ex vivo*

The reporter FLARE Af293-dsRed conidia were generated as above, described under the "Generation of fluorescent *Aspergillus* reporter (FLARE) conidia" section. Bone marrow neutrophils were isolated as described under "Isolation of neutrophils from mouse bone marrow, lungs and bronchoalveolar lavage" (15). Neutrophils were co-incubated with the opsonized FLARE conidia for 3 hours at a 1:1 multiplicity of infection (MOI). Subsequently, the neutrophils were stained using a human anti-Ly6G antibody (clone 1A8, BioLegend, Cat# 127628) and analyzed by flow cytometry to define Ly6G$^+$ neutrophils that are associated with conidia (dsRed/mRFP$^-$ AF633$^+$ or dsRed/mRFP$^+$ AF633$^+$), neutrophils that contain live (dsRed$^+$ AF633$^+$ or mRFP$^+$ AF633$^+$) or killed conidia (dsRed$^-$ AF633$^+$ or mRFP$^-$ AF633$^+$), and bystander neutrophils that have not engaged with conidia (dsRed$^-$ or mRFP$^-$ and AF633$^-$). Flow cytometry was performed using a 5-laser BD LSR Fortessa II (BD Biosciences, Franklin Lakes, NJ). Data were collected using FACS Diva and analyzed using FlowJo (BD Biosciences, Franklin Lakes, NJ).

Aspergillus fumigatus **hyphal damage by mouse neutrophils**

Mouse neutrophils were used in the hyphal killing assay as specified above, under the "*Aspergillus fumigatus* hyphal damage by neutrophils" section. After hypotonic lysis of neutrophils, hyphae were washed with deionized water and incubated with 1X alamarBlue^{TM} (Invitrogen, Cat# DAL 1100) in 37°C incubator with 5% CO_2 for 18 hours. alamarBlue^{TM} fluorescence was recorded using a microplate reader (Agilent, BioTek Synergy H1) and percentage hyphal damage was calculated relative to non-neutrophil-containing wells.

Flow cytometric evaluation of ROS production by mouse lung neutrophils

Mouse lung neutrophils from *Aspergillus* infected mice were isolated at day 2 post-infection, as described above. A dihydrorhodamine-based ROS assay was employed to assess ROS production, as described above. Briefly, $2.5x10^5$ neutrophils were stimulated with opsonized live *Af* conidia ($1x10^8$/ml), opsonized heat-killed *Af* conidia ($1x10^8$/ml), opsonized zymosan (1 mg/ml) (MilliporeSigma, Cat# Z4250), phorbol-12-myristate-13-acetate (PMA) (MilliporeSigma, Cat# P8139) or control in the presence of dihydrorhodamine 123 (Invitrogen, Cat# D632) added to a final concentration of 125 µM. After 30 minutes of stimulation, the cells were put on ice to stop the reaction, and were stained by adding anti-mouse CD11b (clone M1/70, BioLegend, Cat# 101226) and anti-mouse Ly6G (clone 1A8, BioLegend, Cat# 127616). The fluorescence was immediately measured in the FITC channel via flow cytometry by gating on the $Ly6G^+CD11b^+$ neutrophils. Neutrophils were acquired on a BD LSR Fortessa II instrument using BD FACS Diva acquisition software and analyzed using FlowJo software (BD Biosciences, Franklin Lakes, NJ).

Measurement of mouse neutrophil degranulation in response to *Aspergillus fumigatus*

The supernatants of mouse bone-marrow neutrophils co-incubated with preformed hyphae, were harvested and used for downstream enzyme-linked immunosorbent-assay (ELISA) experiments to measure neutrophil degranulation response against *Aspergillus* hyphae. We used the mouse myeloperoxidase DuoSet ELISA kit (R&D Systems, Cat# DY3667) according to kit instructions to measure myeloperoxidase (MPO) from the supernatants taken after mouse neutrophil-*Aspergillus* hyphae co-culture. Absorbance of

the ELISA plates was read at 450 nm wavelength with 570 nm wavelength correction using a microplate reader (Agilent, BioTek Synergy H1).

References

1. Nierman WC, Pain A, Anderson MJ, Wortman JR, Kim HS, Arroyo J, et al. Genomic sequence of the pathogenic and allergenic filamentous fungus Aspergillus fumigatus. *Nature.* 2005;438(7071):1151-6.
2. Jhingran A, Mar KB, Kumasaka DK, Knoblaugh SE, Ngo LY, Segal BH, et al. Tracing conidial fate and measuring host cell antifungal activity using a reporter of microbial viability in the lung. *Cell Rep.* 2012;2(6):1762-73.
3. Kubodera T, Yamashita N, and Nishimura A. Transformation of Aspergillus sp. and Trichoderma reesei using the pyrithiamine resistance gene (ptrA) of Aspergillus oryzae. *Biosci Biotechnol Biochem.* 2002;66(2):404-6.
4. Miller BL, Miller KY, and Timberlake WE. Direct and indirect gene replacements in Aspergillus nidulans. *Mol Cell Biol.* 1985;5(7):1714-21.
5. Willger SD, Puttikamonkul S, Kim KH, Burritt JB, Grahl N, Metzler LJ, et al. A sterol-regulatory element binding protein is required for cell polarity, hypoxia adaptation, azole drug resistance, and virulence in Aspergillus fumigatus. *PLoS Pathog.* 2008;4(11):e1000200.
6. Jones JT, Liu KW, Wang X, Kowalski CH, Ross BS, Mills KAM, et al. Aspergillus fumigatus Strain-Specific Conidia Lung Persistence Causes an Allergic Broncho-Pulmonary Aspergillosis-Like Disease Phenotype. *mSphere.* 2021;6(1).
7. Gupta S, Nakabo S, Blanco LP, O'Neil LJ, Wigerblad G, Goel RR, et al. Sex differences in neutrophil biology modulate response to type I interferons and immunometabolism. *Proc Natl Acad Sci U S A.* 2020;117(28):16481-91.
8. Durinck S, Moreau Y, Kasprzyk A, Davis S, De Moor B, Brazma A, et al. BioMart and Bioconductor: a powerful link between biological databases and microarray data analysis. *Bioinformatics.* 2005;21(16):3439-40.
9. Durinck S, Spellman PT, Birney E, and Huber W. Mapping identifiers for the integration of genomic datasets with the R/Bioconductor package biomaRt. *Nat Protoc.* 2009;4(8):1184-91.
10. Robinson MD, McCarthy DJ, and Smyth GK. edgeR: a Bioconductor package for differential expression analysis of digital gene expression data. *Bioinformatics.* 2010;26(1):139-40.
11. El-Benna J, and Dang PM. Analysis of protein phosphorylation in human neutrophils. *Methods Mol Biol.* 2007;412:85-96.
12. Belambri SA, Hurtado-Nedelec M, Senator A, Makni-Maalej K, Fay M, Gougerot-Pocidalo MA, et al. Phosphorylation of p47phox is required for receptor-mediated NADPH oxidase/NOX2 activation in Epstein-Barr virus-transformed human B lymphocytes. *Am J Blood Res.* 2012;2(3):187-93.
13. Schindelin J, Arganda-Carreras I, Frise E, Kaynig V, Longair M, Pietzsch T, et al. Fiji: an open-source platform for biological-image analysis. *Nat Methods.* 2012;9(7):676-82.
14. Jennings RT, and Knaus UG. Rho family and Rap GTPase activation assays. *Methods Mol Biol.* 2014;1124:79-88.
15. Wishart AL, Swamydas M, and Lionakis MS. Isolation of Mouse Neutrophils. *Curr Protoc.* 2023;3(9):e879.

16. Drummond RA, Collar AL, Swamydas M, Rodriguez CA, Lim JK, Mendez LM, et al. CARD9-Dependent Neutrophil Recruitment Protects against Fungal Invasion of the Central Nervous System. *PLoS Pathog.* 2015;11(12):e1005293.
17. Lionakis MS, Lim JK, Lee CC, and Murphy PM. Organ-specific innate immune responses in a mouse model of invasive candidiasis. *J Innate Immun.* 2011;3(2):180-99.
