# Supplementary material for: BTK drives neutrophil activation for sterilizing antifungal immunity
Source: J Clin Invest. 2024 May 2;134(12):e176142. doi: 10.1172/JCI176142 (PMC11178547; doi:10.1172/JCI176142)

Full unedited blot/gel for Figure 5A

p40^{phox} (phospho T154)

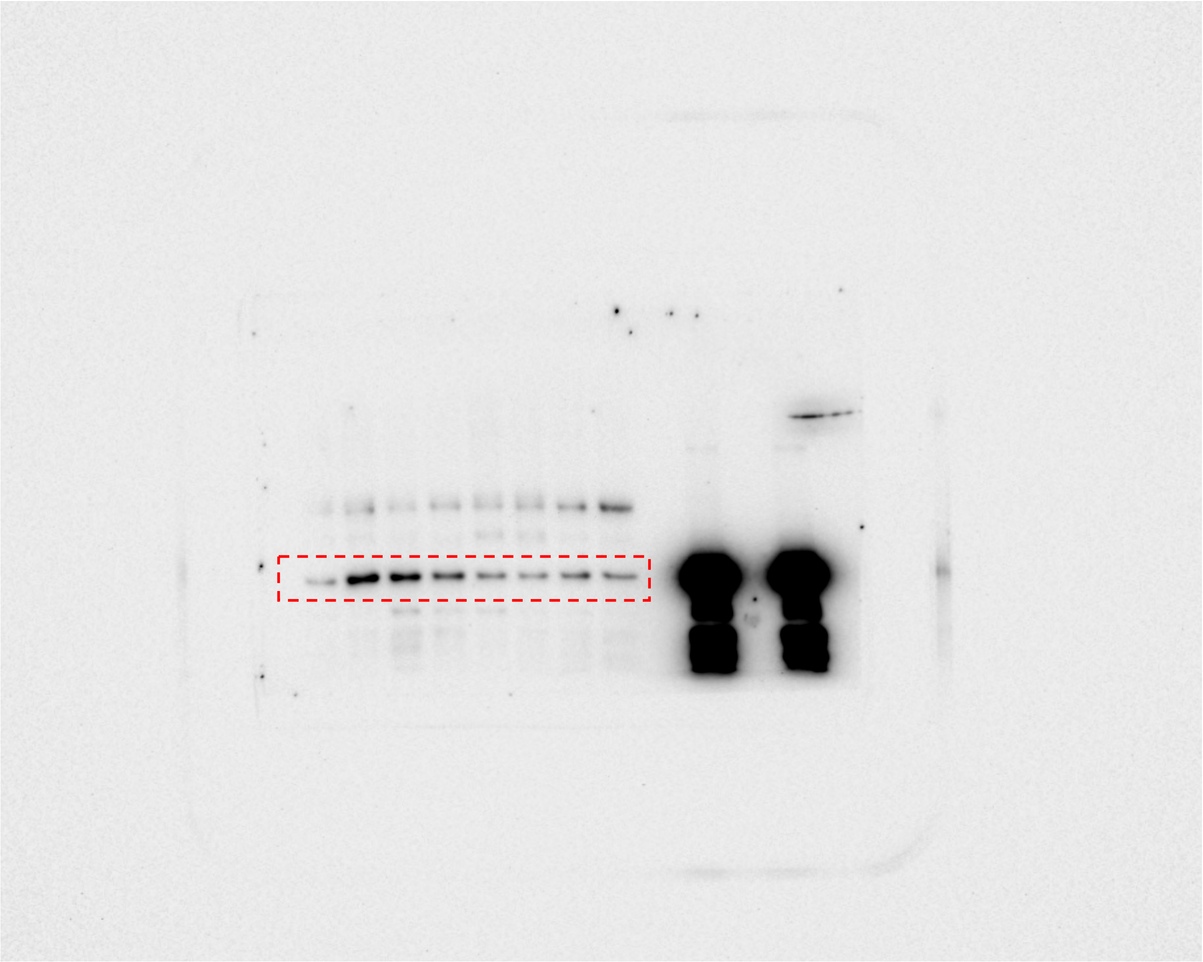

p40^{phox} (total)

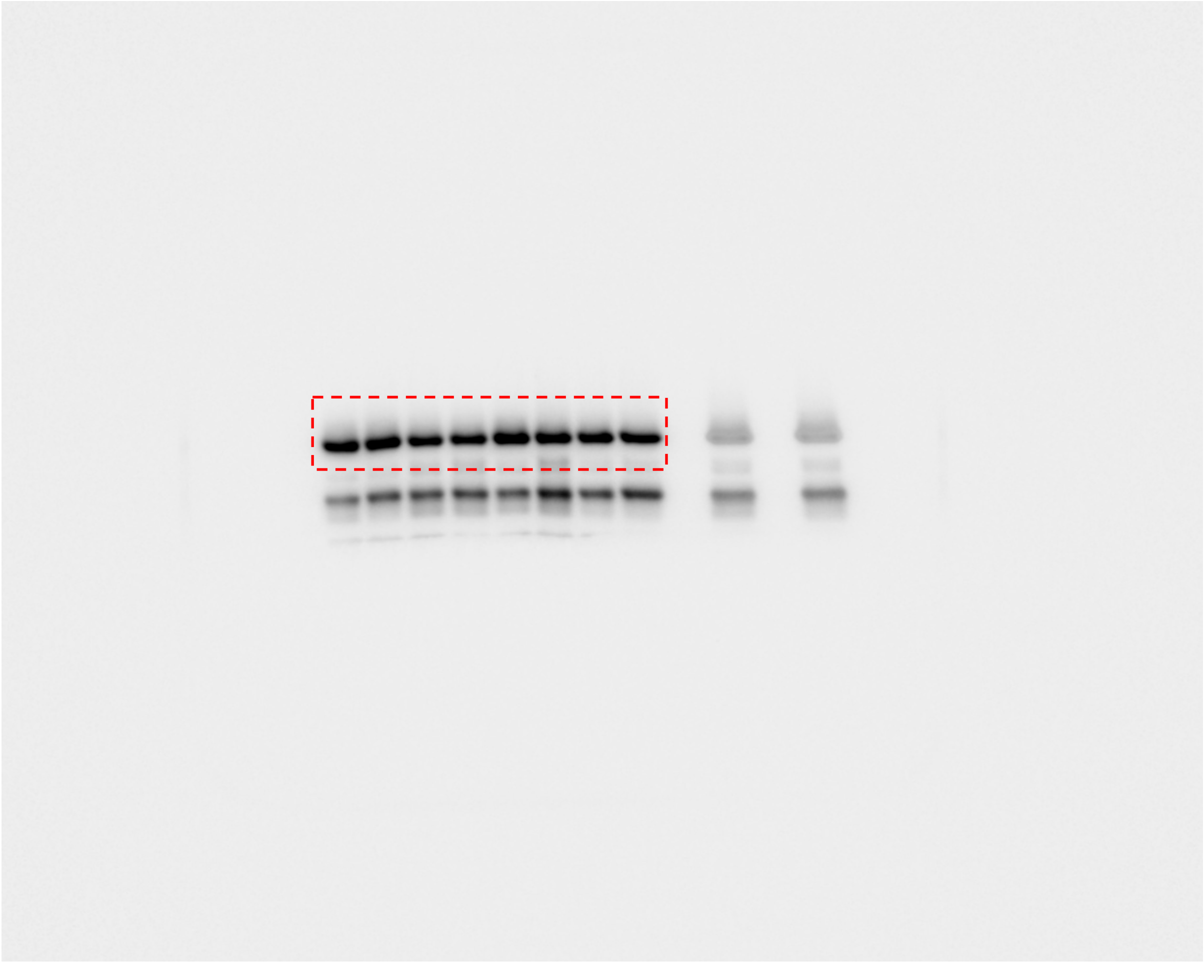

Full unedited blot/gel for Figure 5B

p40^{phox} (phospho T154)

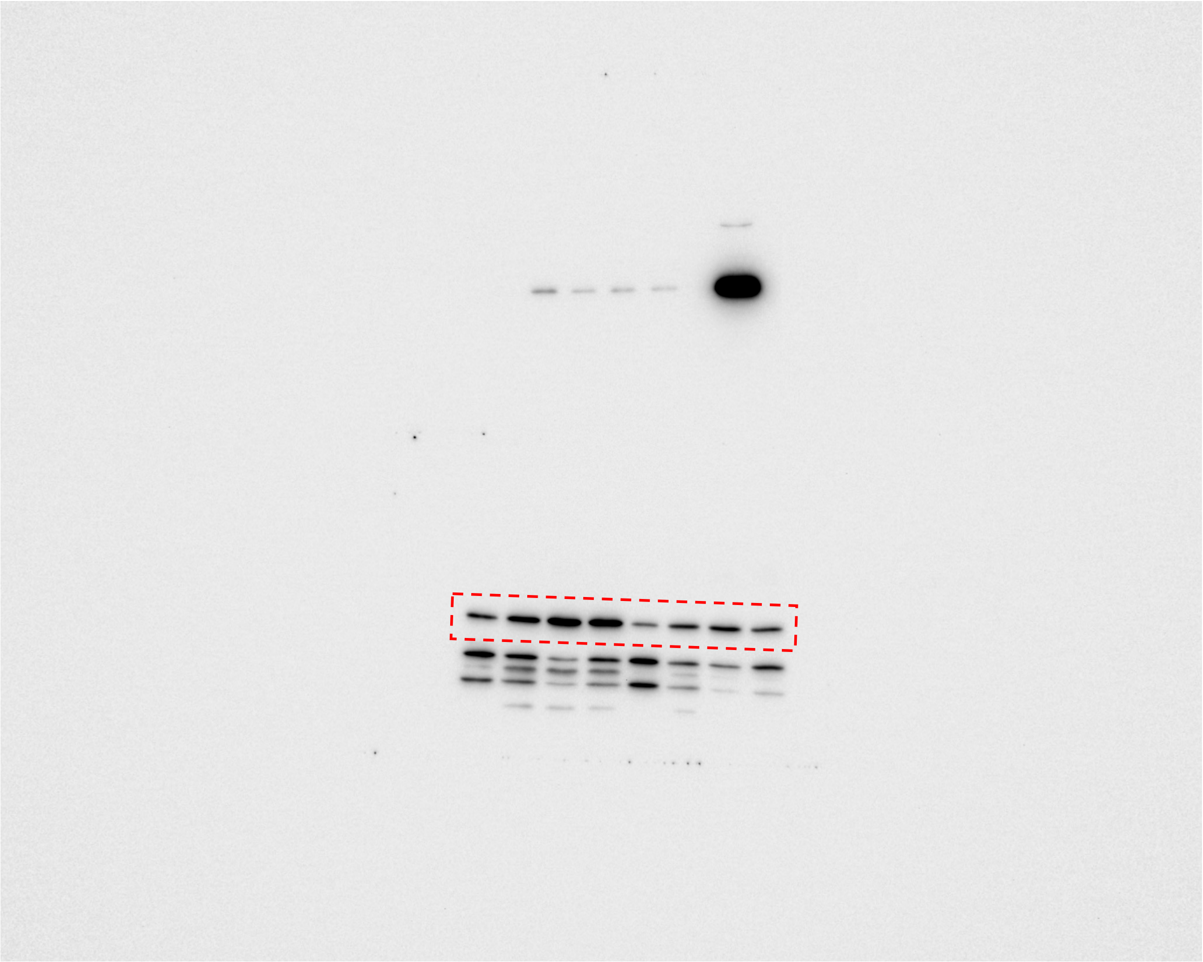

p40^{phox} (total)

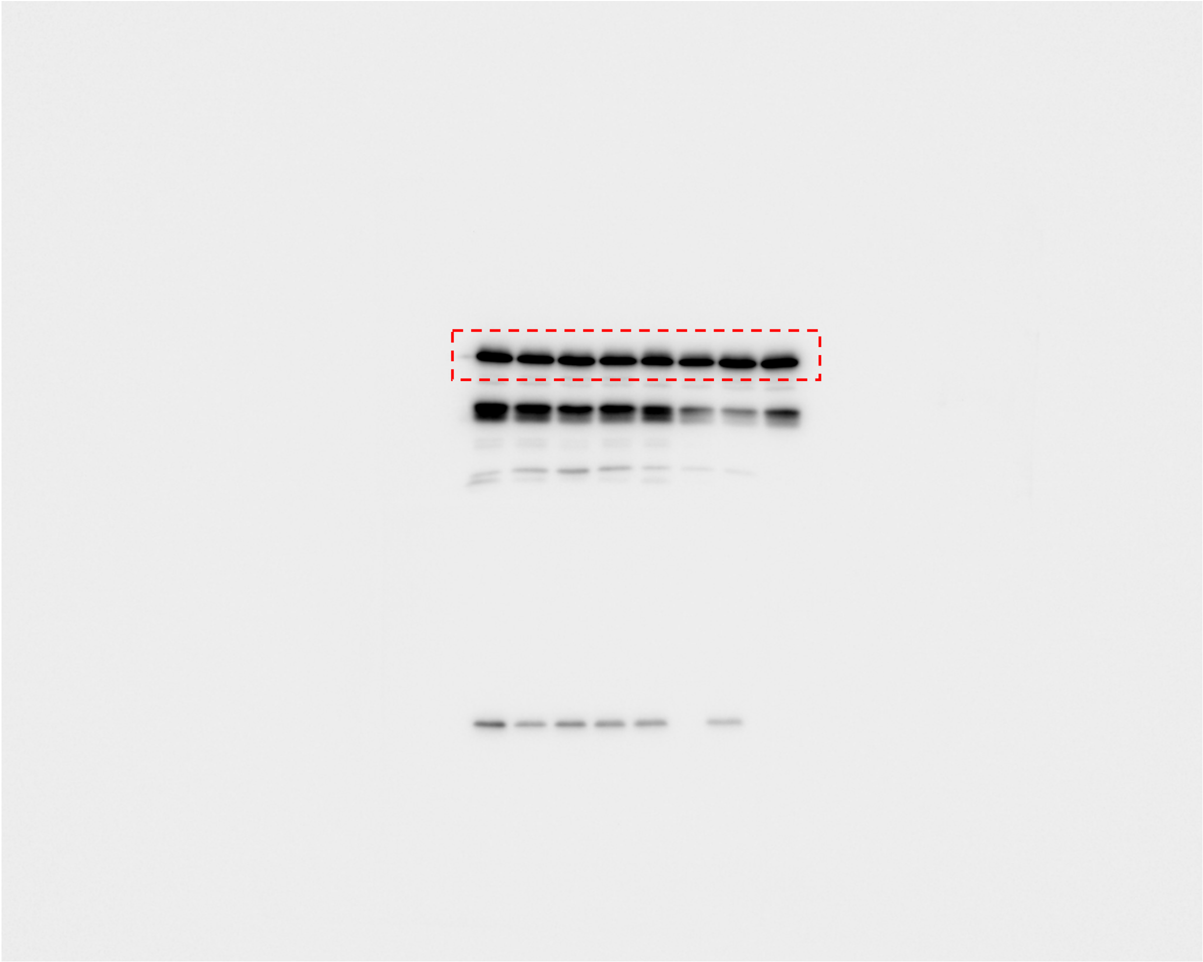

Full unedited blot/gel for Figure 5C

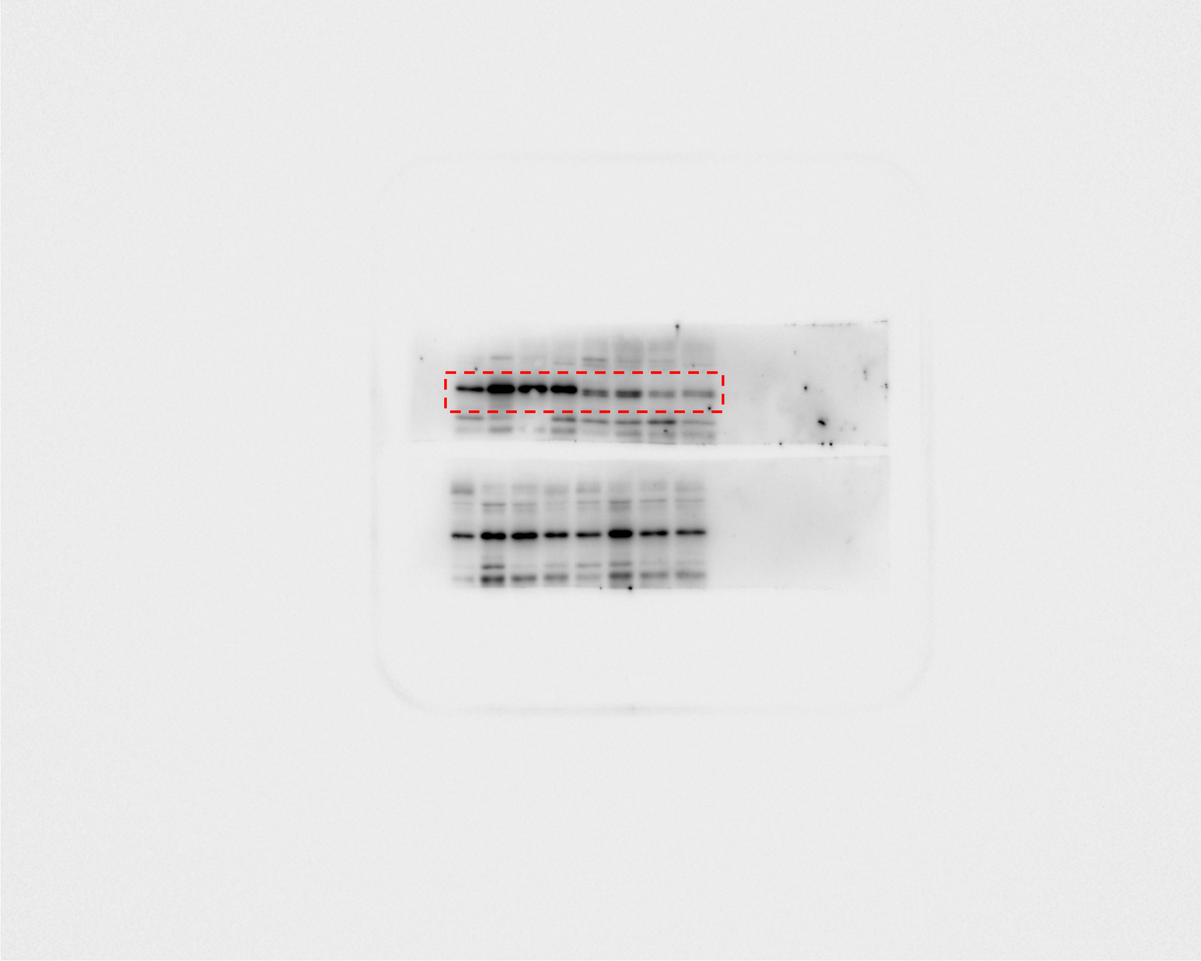

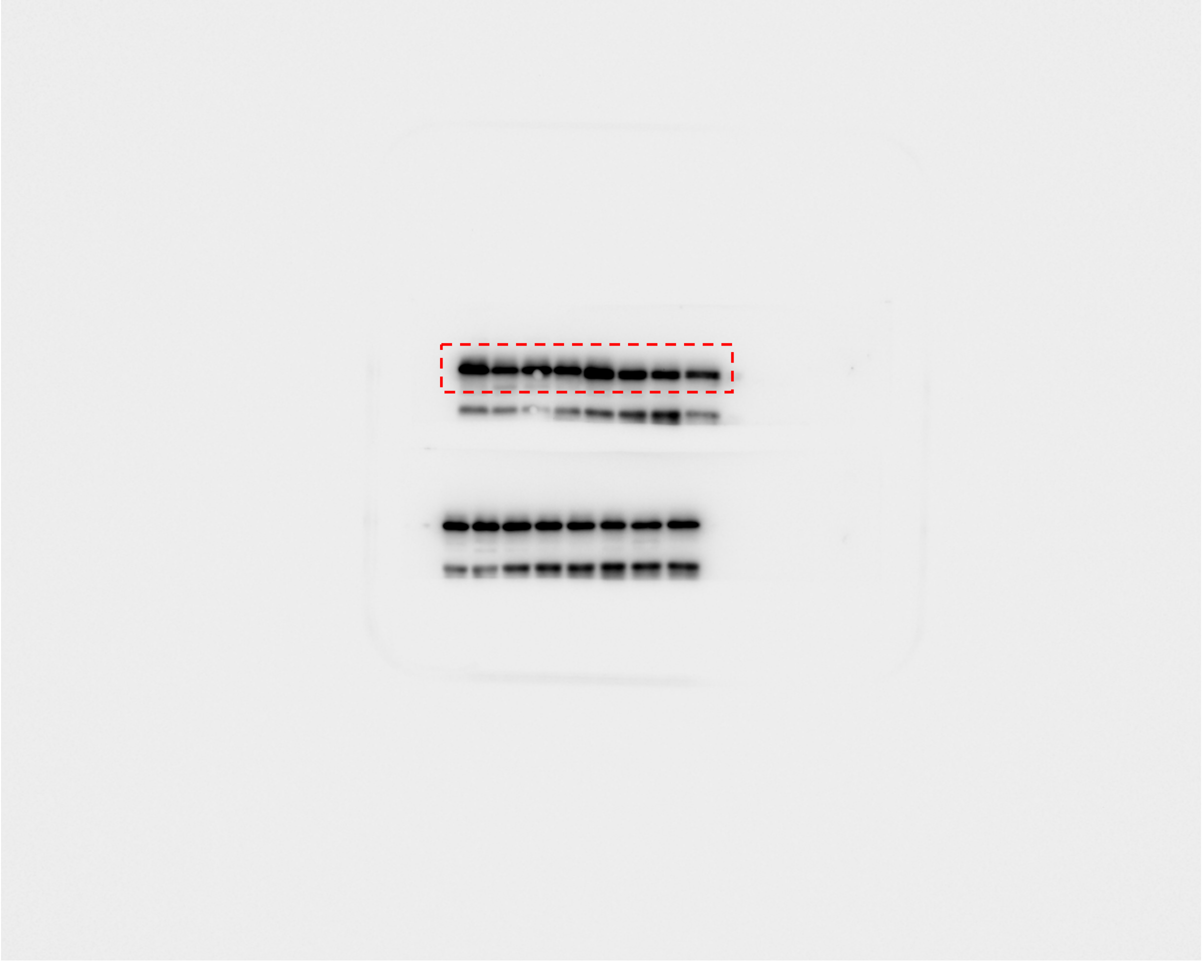

Full unedited blot/gel for Figure 5D

RAC2-GTP for GDP, GTPyS (left upper blot)

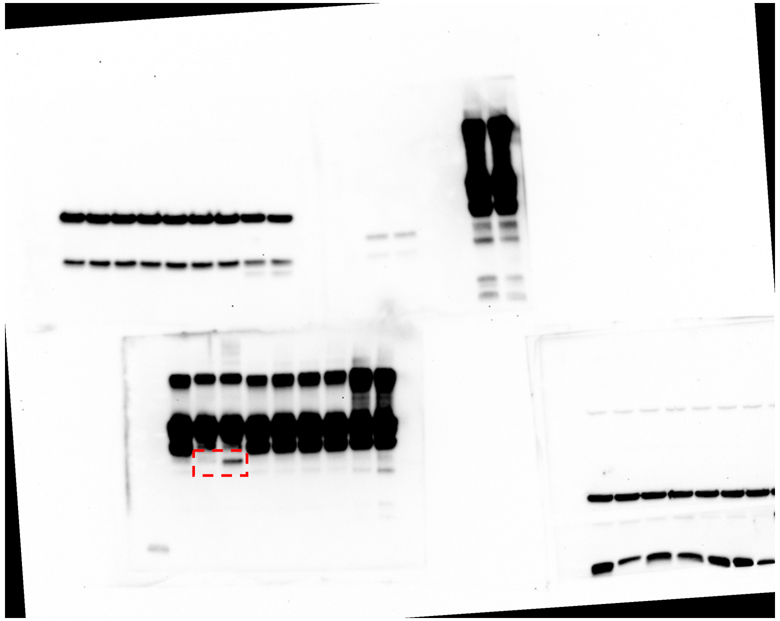

RAC2-GTP for unstim, HBSS, vehicle and ibrutinib (right upper blot)

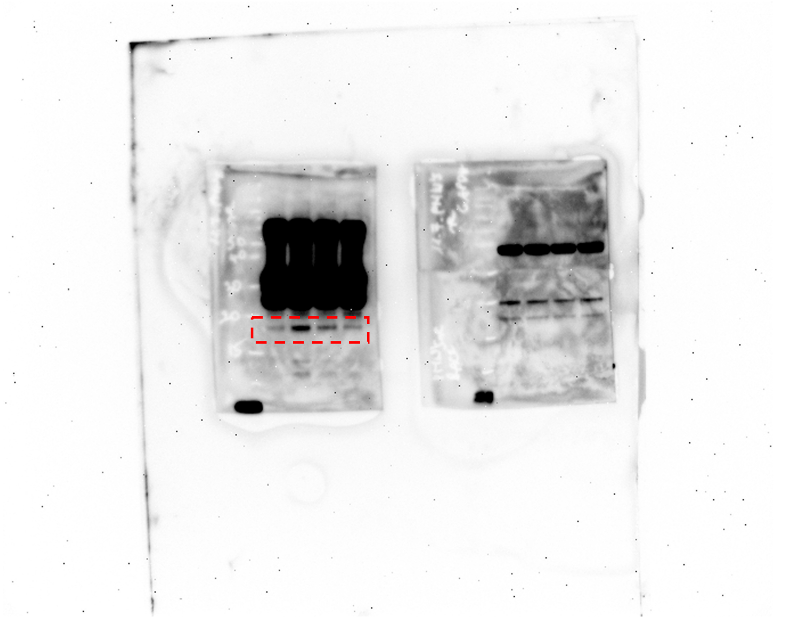

RAC2 (total) for GDP, GTPyS (left lower blot)

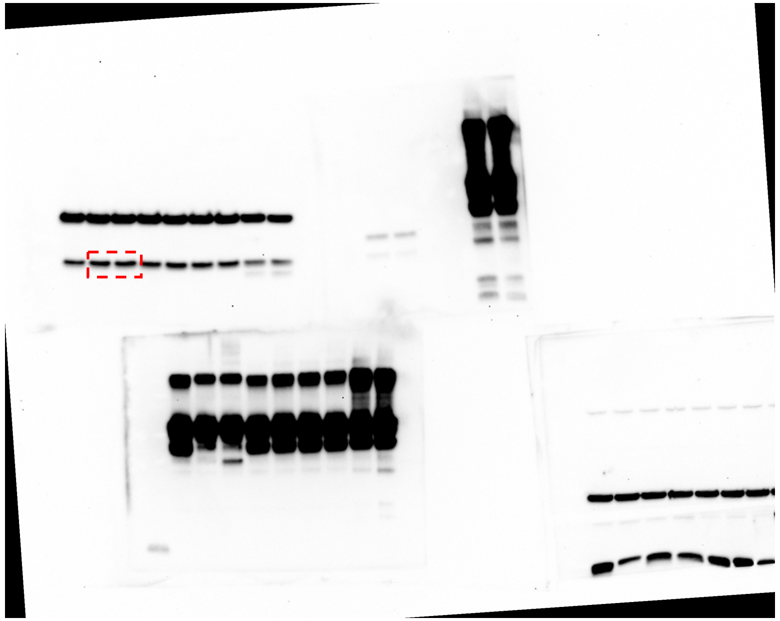

RAC2 (total) for unstim, HBSS, vehicle and ibrutinib (right lower blot)

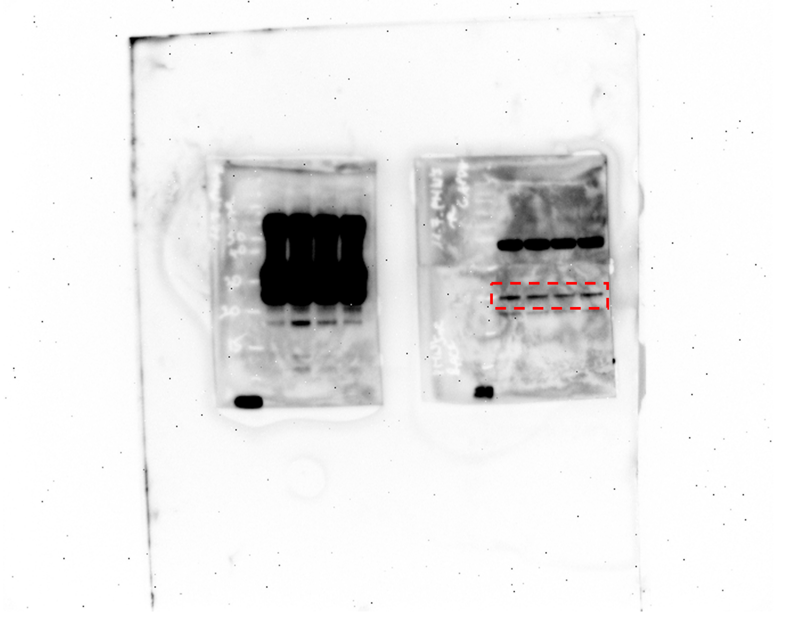

Full unedited blot/gel for Figure 7D

p40^{phox} (phospho T154)

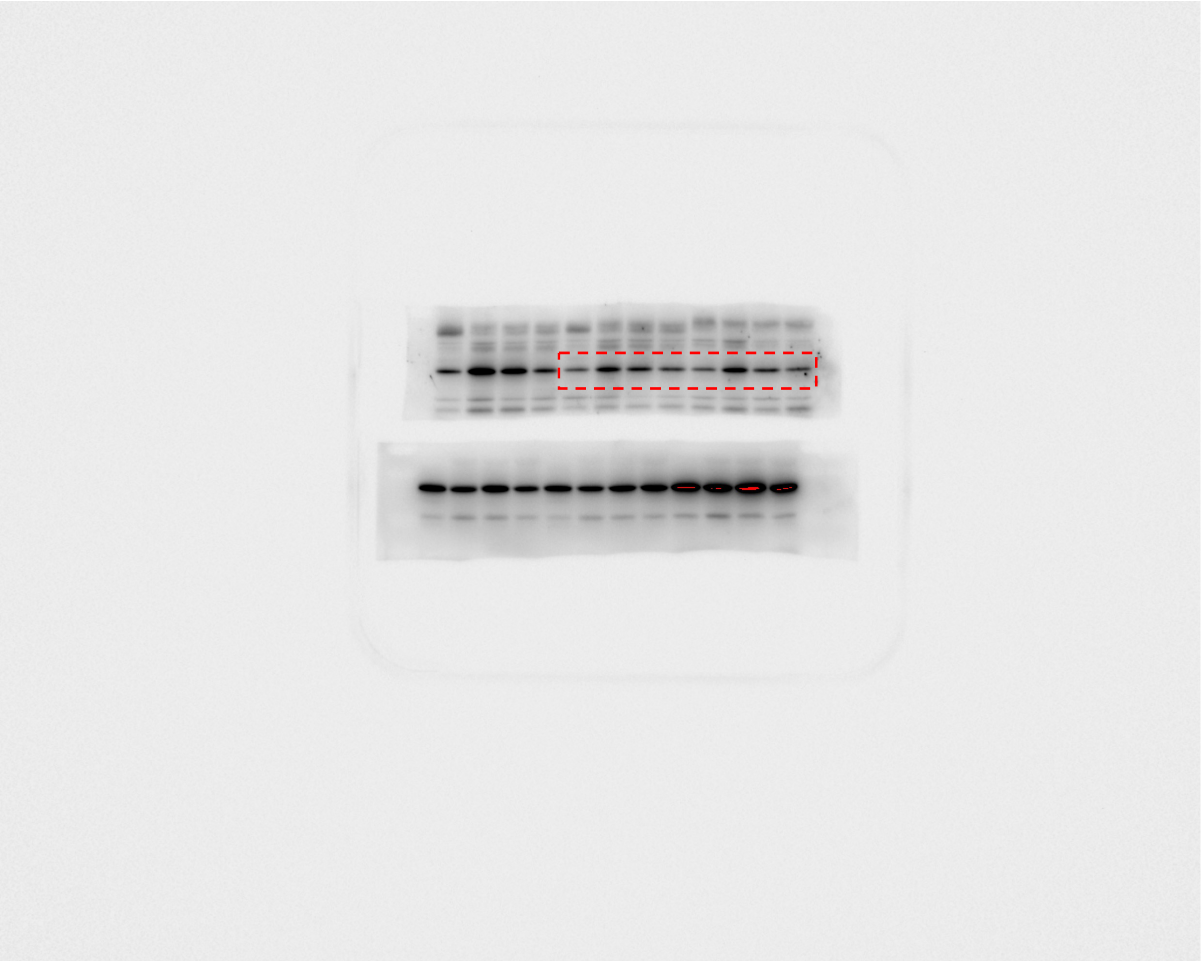

p40^{phox} (total)

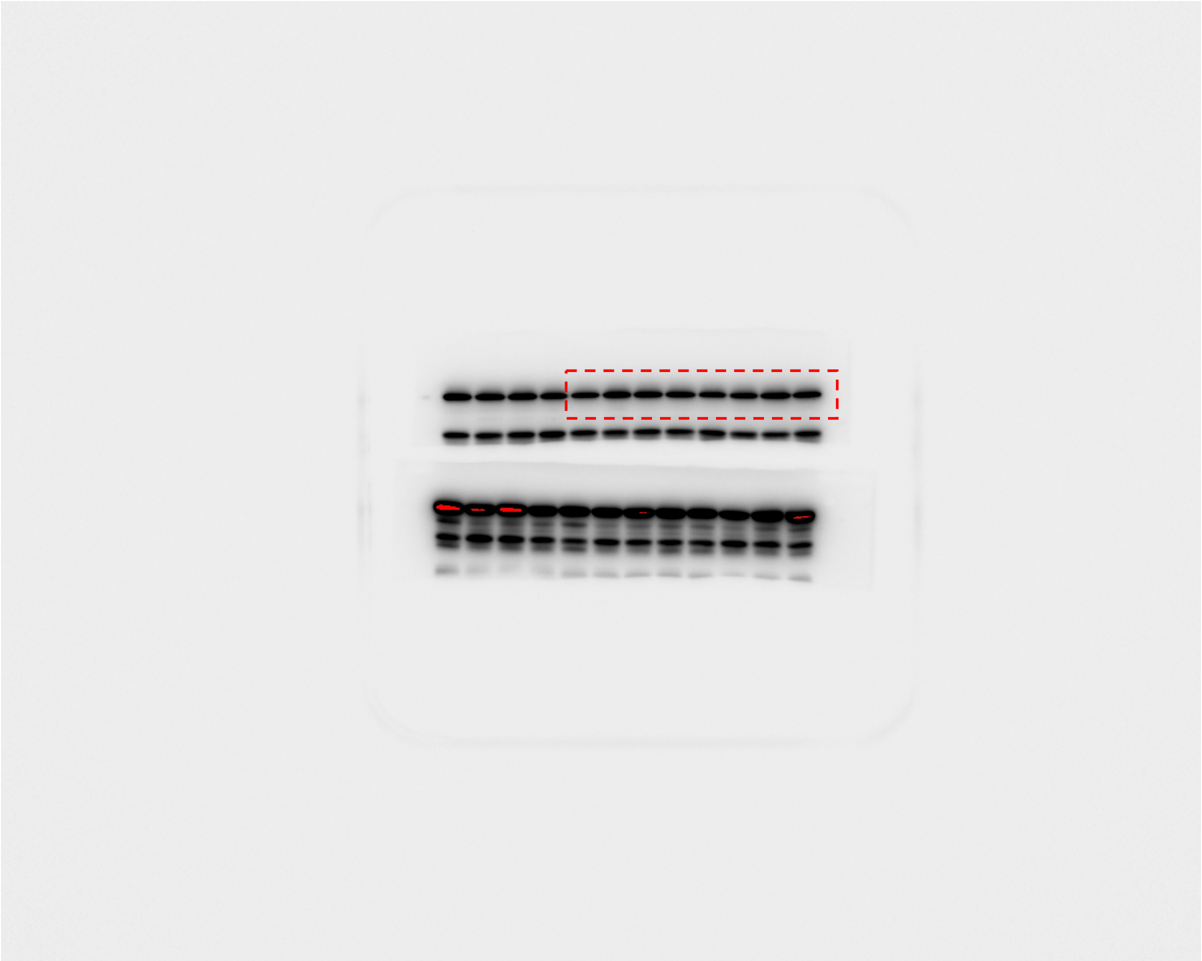

Full unedited blot/gel for Figure 7E

p47[phox] (phospho S345)

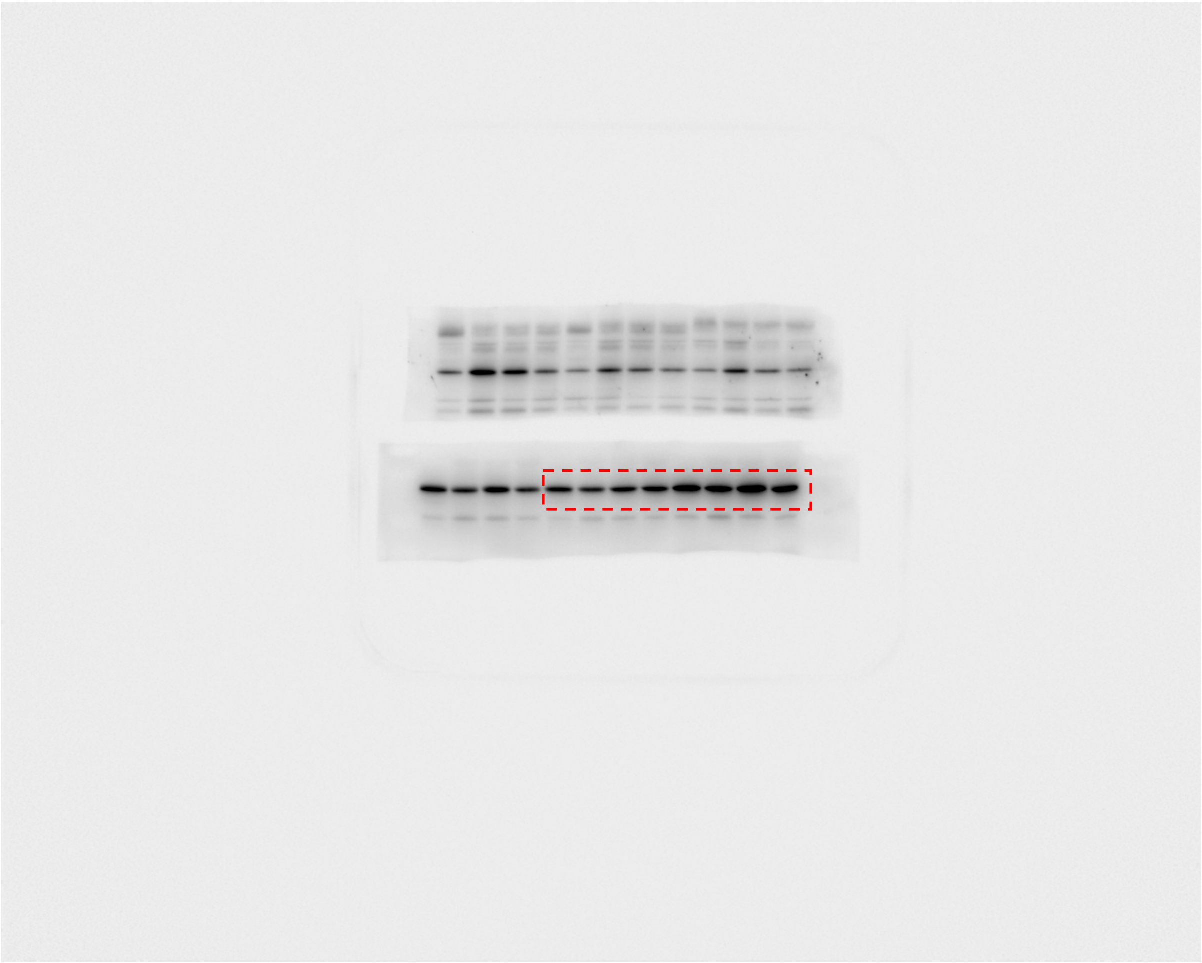

p47[phox] (total)

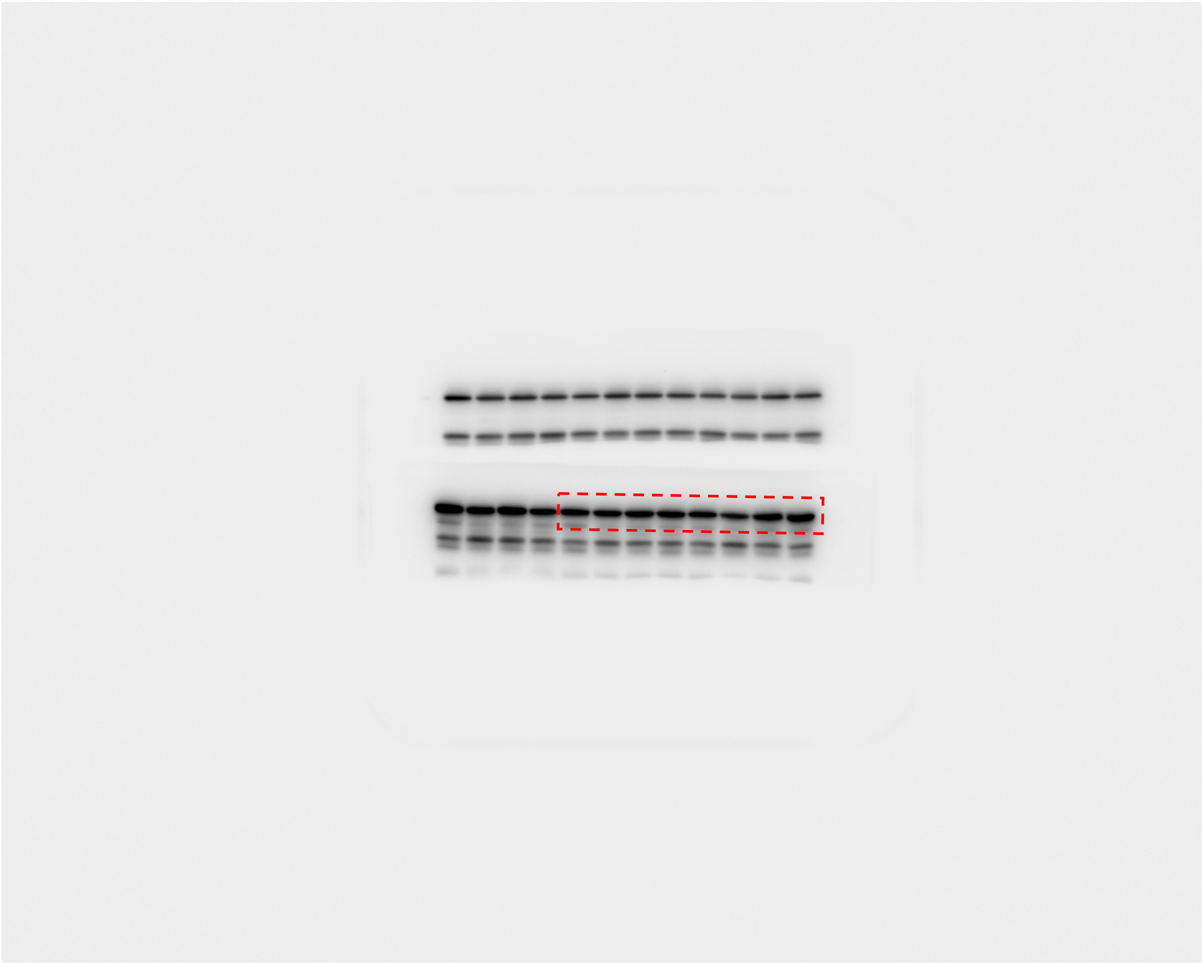

Full unedited blot/gel for Supplemental Figure 5

p40phox (phospho T154)

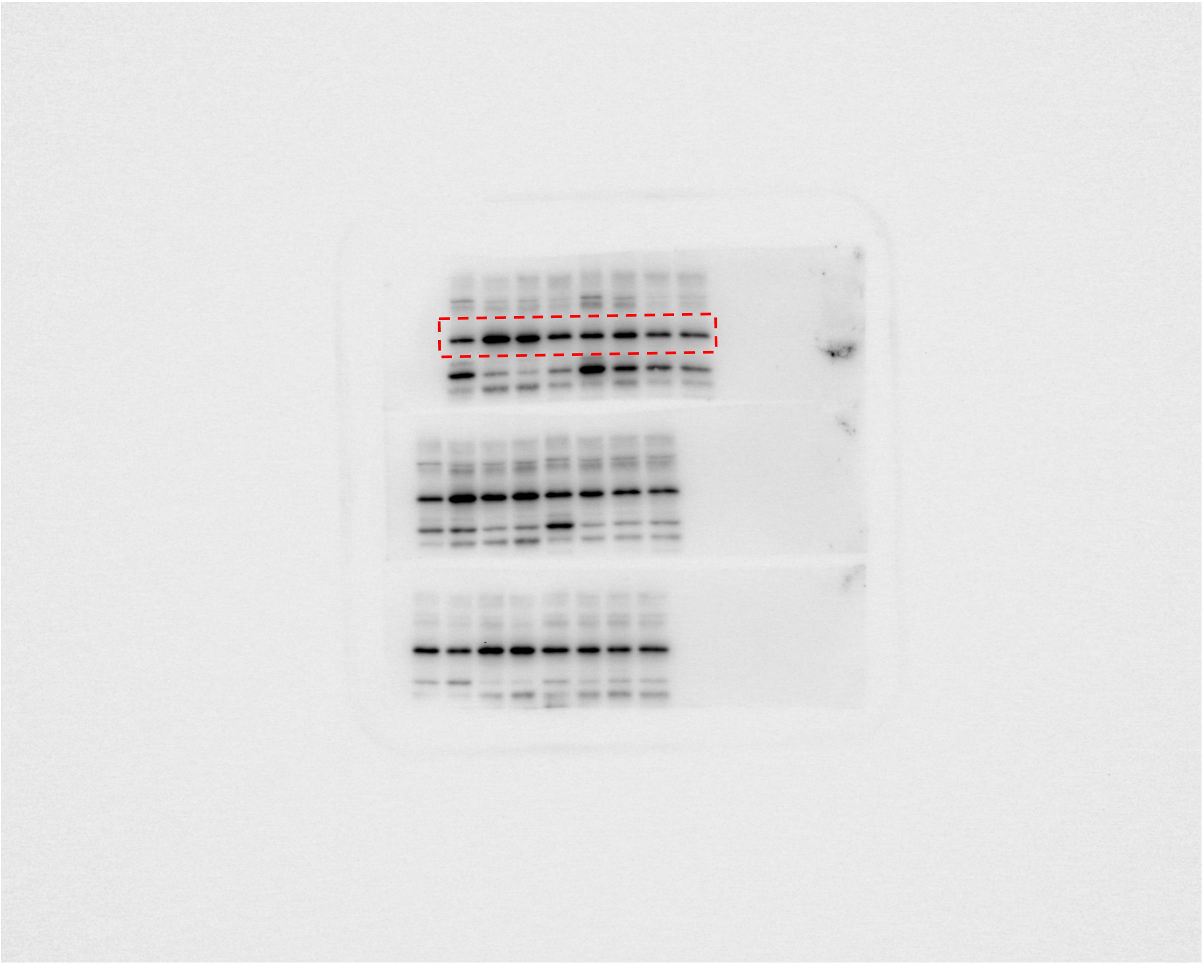

p40phox (total)

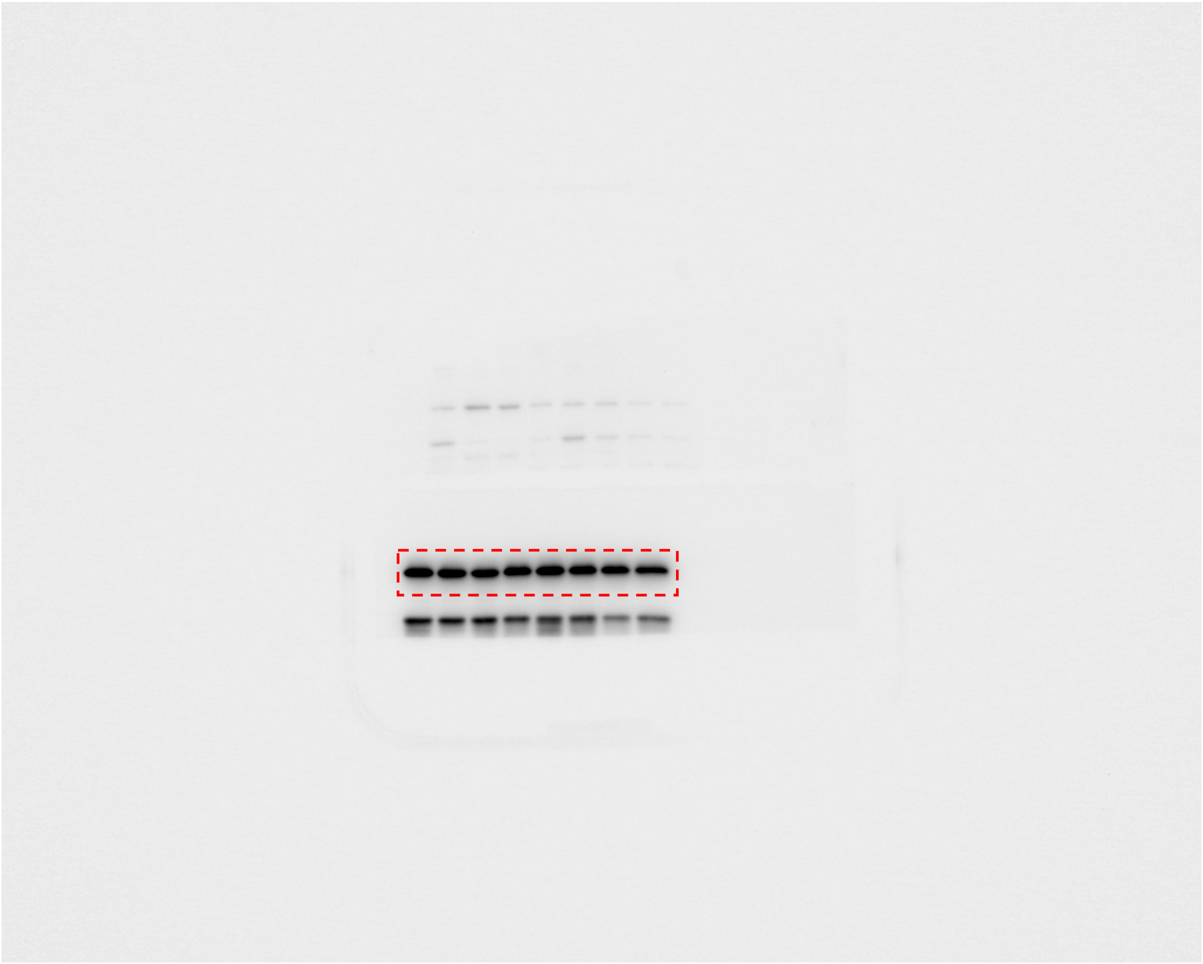

Supplement: Unedited blot and gel images [file jci-134-176142-s068.pdf]
